# Osteoarthritis as a systemic disorder: multi-organ crosstalk in pathogenesis and therapeutic targeting

**DOI:** 10.3389/fimmu.2026.1871461

**Published:** 2026-07-08

**Authors:** Tao Shu, Xiaobin Shang, Yan Zhou

**Affiliations:** Department of Orthopedics, Renmin Hospital of Wuhan University, Wuhan, China

**Keywords:** osteoarthritis, gut-brain-liver-kidney axis, systemic inflammation, central sensitization, metabolic regulation

## Abstract

While osteoarthritis (OA) has long been viewed primarily as a localized, mechanically-driven joint disorder, emerging evidence suggests that systemic factors may play a significant modulating role in its pathogenesis. This review presents the “Gut-Brain-Liver-Kidney axis” as a potential regulatory framework to explore a conceptual shift towards a systemic perspective on this traditionally localized disease. Available evidence is synthesized to clarify how gut microbiota dysbiosis and its metabolites contribute to systemic inflammation and disrupt joint homeostasis through specific pathways, such as the GUDCA-FXR-GLP-1 axis. Bacterial extracellular vesicles are further highlighted as essential nanoscale messengers facilitating communication between the gut and joints. Extending beyond gut health, the significant impact of central sensitization and neuroendocrine dysregulation in the brain is investigated as a key driver of chronic pain perception—a phenomenon often disproportionate to observable structural damage. Unlike mechanisms that directly cause cartilage breakdown, central sensitization primarily modulates pain experience and can secondarily influence disease progression by promoting maladaptive behaviors (e.g., reduced mobility). The liver’s involvement is also analyzed, particularly its disorders related to iron and lipid metabolism that promote chondrocyte ferroptosis. Furthermore, the analysis addresses how renal dysfunction intensifies OA by impairing vitamin D metabolism and leading to the accumulation of uremic toxins, such as indoxyl sulfate. By integrating these interconnected systemic pathways, a complex network of potential novel therapeutic targets is revealed. Consequently, innovative strategies aimed at these axes are outlined, including the use of probiotics, vagus nerve stimulation, FGF21, GalNAc-siRNA, and vitamin D supplementation. This perspective encourages moving beyond symptom management toward mechanism-based, multi-targeted strategies. Key unanswered questions are outlined and priorities for future research and clinical translation in this evolving field are proposed.

## 1 Introduction

Osteoarthritis (OA) is a degenerative disease of articular cartilage and has increasingly been recognized as a systemic disorder that involves multiple organs through complex regulatory networks (1). Although its etiology remains not fully understood, its progression is a chronic pathological process characterized by cartilage degradation, synovitis, and osteophyte formation (2). As the most prominent symptom of OA, pain severely influences patients’ quality of life (3). OA affects over 595 million individuals worldwide, resulting in a substantial economic burden on individuals, families, and national healthcare systems. The pathogenesis of OA is traditionally understood as multifactorial, involving a complex interplay of mechanical and biological factors. However, the underlying mechanisms remain incompletely understood, and there are currently no effective pharmacological treatments available to ameliorate this disease. The limitations of conventional therapies, such as nonsteroidal anti-inflammatory drugs (NSAIDs) and joint replacement, are that they primarily alleviate symptoms rather than reverse the progression of the disease (4). It is important to note that the prevailing understanding of OA remains centered on biomechanical overload and local joint pathology as the primary drivers. The extent to which systemic factors actively initiate the disease, as opposed to merely modulating its progression following local onset, is an area of active debate. With this perspective in mind, this review does not assert a definitive paradigm shift, but rather aims to critically synthesize the emerging evidence for multi-organ involvement, proposing an integrative framework for further investigation.

With advancing age as the primary risk factor, additional contributors to OA include trauma, obesity, inflammation, metabolic disturbances, and genetic predisposition. Studies have demonstrated a significant association between OA and patients who present with metabolic syndrome, which encompasses conditions such as obesity and diabetes, as well as other systemic diseases (5).

Recent studies have increasingly demonstrated that systemic factors, including gut microbiota, liver and kidney function, and neural regulation, play a significant role in the progression of OA (6–9). These findings collectively depict a integrative perspective of OA as a systemic disease, suggesting new avenues for therapeutic strategies that target systemic pathways rather than local joint pathology alone. Despite the rapid growth of related research, there is a lack of systematic organization, comparison, and integration of multi-organ regulatory networks in this field.

Therefore, this review is structured around three core objectives to refine its central theme. First, to comprehensively synthesize the emerging evidence for each component of the proposed “Gut-Brain-Liver-Kidney axis”, detailing the specific molecular pathways — including gut microbial metabolites (SCFAs, IPA, GUDCA), bacterial extracellular vesicles, central sensitization and neuroendocrine regulation, hepatic ferroptosis, as well as renal vitamin D and uremic toxin metabolism — that mechanistically link each organ to OA pathogenesis. Second, to critically evaluate the level of evidence (from preclinical models to human studies) supporting a causal versus modulatory role for each systemic axis, while explicitly acknowledging current knowledge gaps, controversies, and the prevailing uncertainty regarding whether systemic factors initiate OA or merely modulate its progression after local onset. Third, to outline how these interconnected systemic pathways converge into a complex regulatory network, thereby revealing multi-target therapeutic opportunities (e.g., probiotics, vagus nerve stimulation, FGF21, GalNAc-siRNA, vitamin D supplementation) that extend beyond conventional symptom management. Ultimately, we aim to provide a conceptual framework that supports a broader, systems-oriented approach to OA research and treatment — moving from a localized, symptom-management strategy toward a systemic, disease-modifying paradigm.

## Methods

### Search strategy and selection criteria

This review was conducted following the methodological guidance for scoping and narrative reviews, aiming to synthesize the emerging evidence on multi-organ crosstalk in osteoarthritis pathogenesis. A systematic literature search was performed across three electronic databases: PubMed, Web of Science, and Scopus. The search strategy combined keywords and Medical Subject Headings (MeSH) terms related to osteoarthritis and systemic organ systems, using Boolean operators. The core search string was: (“osteoarthritis” OR “OA” OR “joint degeneration”) AND (“gut microbiota” OR “dysbiosis” OR “short-chain fatty acids” OR “bile acids” OR “central sensitization” OR “brain-joint” OR “liver” OR “ferroptosis” OR “kidney” OR “vitamin D” OR “uremic toxins” OR “systemic inflammation”).

### Inclusion and exclusion criteria

We included peer-reviewed original research articles (including randomized controlled trials, cohort studies, cross-sectional studies, case-control studies), preclinical studies (*in vivo* animal models and *in vitro* mechanistic studies), and systematic reviews/meta-analyses published in English. Studies were considered eligible if they explicitly investigated associations, causal relationships, or mechanistic pathways between at least one extra-articular organ system (gut, brain, liver, kidney) and osteoarthritis pathogenesis, pain, or progression. We excluded conference abstracts, editorials, opinion pieces without primary data, case reports with fewer than five patients, and studies focusing exclusively on joint-localized pathology (e.g., cartilage biomechanics without any systemic measurement). Studies investigating other forms of arthritis (e.g., rheumatoid arthritis) were included only if they provided mechanistic insights relevant to OA.

### Data extraction and synthesis

For each included study, we extracted: (1) study design and sample characteristics (human/animal/cell line), (2) the specific organ axis investigated, (3) key molecular pathways or mediators, (4) direction of effect (protective vs. pathogenic), and (5) limitations as reported by authors. The findings were synthesized narratively around the proposed “Gut-Brain-Liver-Kidney axis”, with separate sections for each organ system. Given the heterogeneity of study designs and outcome measures, we did not perform a quantitative meta-analysis but instead summarized evidence levels descriptively as “preclinical (*in vitro*/animal)”, “human observational (cross-sectional/cohort)”, “human genetic (Mendelian randomization)”, or “clinical interventional (RCT)”, as indicated in **Tables 1** and **2**. Causal language (e.g., “drives”, “promotes”) is used only when supported by interventional data (animal or human) or Mendelian randomization; otherwise, we use associative language (e.g., “is associated with”, “may contribute to”).

**Table 1:** Mechanisms of the gut-brain-liver-kidney axis in OA.

Organ axis	Core mechanism	Key pathways/molecules	Effect	Reference
Gut−Joint axis	Dysbiosis of gut microbiota, impairment of intestinal barrier function	LPS−TLR4−NF-κB pathway;ZO-1, Occludin	Induce systemic low-grade inflammation, exacerbate joint inflammation and cartilage degradation	(16, 17, 19, 22–27)
	Gut microbiota metabolism regulates SCFAs and IPA	SCFAs−inhibit HDAC/activate GPCR; IPA −AhR−NF-κB pathway	SCFAs exert anti-inflammatory effects and protect cartilage; IPA alleviates chondrocyte inflammation and catabolism	(28–31, 34–38)
	Bile acid signaling axis	*C. bolteae−*GUDCA−intestinal FXR−GLP-1 axis; TGR5−L cell−GLP-1; NF-κB; AMPK	There is a negative correlation between GLP-1 levels and the severity of OA; Protects cartilage, limits inflammation and apoptosis, and promotes bone resorption	(10, 40–45, 47, 49, 50, 183)
	BEVs	LPS, PGs, nucleic acids, and other substances carried by BEVs	Pathogenic bacteria-derived BEVs exert pro-inflammatory effects; probiotic-derived BEVs exert anti-inflammatory effects	(11, 26, 52–59)
Brain−Joint axis	Central Sensitization	microglial activation−CX3CL1/CX3CR1−p38/MAPK pathway	Pain is decoupled from the degree of joint structural damage	(82–85)
	Neuroendocrine Dysregulation	HPA axis; vagus nerve−acetylcholine−α7nAChR	Dysregulation of the HPA axis impairs the inhibition of endogenous inflammation; activation of the vagus nerve can attenuate cartilage degradation	(89, 90, 92, 93)
Liver−Joint axis	IGF-1 Metabolism	IGF-1−PI3K/Akt/MAPK pathway	Inhibit the production of ROS and cell apoptosis to delay the progression of OA	(111–113)
	Iron Metabolism	Hepcidin-ferroportin axis; Fenton reaction−lipid peroxidation; IL-6−hepcidin−iron retention in macrophages−NAFLD-related dyslipidemia−increased PUFA delivery	Promotes chondrocyte death, cartilage degradation, and OA progression; ferroptosis inhibitors and GPX4 overexpression protect against OA in animal models; chondrocyte-specific GPX4 knockout induces OA-like changes	(13, 115, 117, 118, 120, 123, 125)
Kidney−Joint axis	Chronic Kidney Disease and Mineral Metabolism Disorder	FGF23−Wnt/β-catenin pathway; Uremic toxins	Upregulates the expression of MMP13/9 in chondrocytes to promote cartilage degradation; uremic toxins may indirectly affect the joints	(135–137)
	Vitamin D Metabolism	Vitamin D−VDR/RXR−TFEB axis; AMPK/mTOR and PI3K/Akt/mTOR pathways	Promotes chondrocyte autophagy, eliminates damaged organelles, inhibits inflammatory cell death, and protects cartilage	(138–141, 184)

LPS, Lipopolysaccharide; TLR4, Toll-like Receptor 4; ZO-1, Zonula Occludens-1; SCFAs, short-chain fatty acids; HDAC, Histone Deacetylase; GPCR, G Protein-Coupled Receptor; IPA, Indole-3-Propionic Acid; AhR, Aryl Hydrocarbon Receptor; GUDCA, Glycoursodeoxycholic Acid; FXR, Farnesoid X Receptor; GLP-1, Glucagon-like Peptide-1; OA, Osteoarthritis; BEVs, Bacterial Extracellular Vesicles; PGs, Prostaglandins; CX3CL1, Chemokine (C-X3-C motif) ligand 1; CX3CR1, Chemokine (C-X3-C Motif) Receptor 1; HPA, Hypothalamic-Pituitary-Adrenal axis; α7nAChR, α7 Nicotinic Acetylcholine Receptor; IGF-1, Insulin-like Growth Factor 1; ROS, reactive oxygen species; OA, osteoarthritis; NAFLD, Non-alcoholic fatty liver disease; PUFA, Polyunsaturated fatty acid; GPX4, Glutathione Peroxidase 4; FGF23, Fibroblast Growth Factor 23; MMP13/9, Matrix Metalloproteinase 13/9; VDR, Vitamin D Receptor; RXR, Retinoid X Receptor; TFEB, Transcription Factor EB.

**Table 2:** Therapeutic strategies targeting the gut-brain-liver-kidney axis in OA.

Organ axis	Treatment strategy	Evidence level for OA	Effect	Key limitations/null findings	Reference
Gut-Joint axis	Probiotics/Prebiotics	RCTs available (moderate evidence)	Regulates gut microbiota, inhibits systemic and joint inflammation; Ferment to SCFAs, reinforcing intestinal barrie	Small sample sizes; strain-dependent effects; need for larger confirmatory trials	(31, 60–64)
	FMT	Preclinical only (no human RCT for OA)	Corrects gut microbiota dysbiosis, restores intestinal barrier function, and alleviates systemic inflammation	No controlled human trials for OA; ethical and practical limitations for long-term studies	(19, 65, 66, 68)
	Bioactive compounds	Preliminary (mostly preclinical; few small RCTs for symptomatic relief	Multi-target anti-inflammatory and chondroprotective actions;	Poor oral bioavailability for many compounds; Lack of head-to-head RCTs versus standard OA therapies	(71, 72, 74, 77)
Brain-Joint axis	Pharmacological intervention (central sensitization)	RCTs available for pain (but not disease modification)	Reduce neuronal hyperexcitability; enhance descending inhibition; modulate endocannabinoid system	Evidence for OA-specific prescribing is limited; side effect concerns	(96–100)
	Pharmacological intervention (Neuroendocrine)	Limited evidence/case series (no OA-specific RCT)	Restores HPA axis rhythm; reduces sympathetic tone; may influence joint inflammation via autonomic regulation	Only applicable to specific subpopulations (e.g., adrenal insufficiency); lack of OA-focused trials	(102–104)
	Non-pharmacological intervention	RCTs available (moderate to strong for exercise)	Modulates cortical excitability; alters pain-related cognitive/behavioral patterns	Variable efficacy across modalities; requires personalized approach	(105–109)
Live-Joint axis	Hepato-derived factor therapy	Preclinical only	Promotes chondrocyte autophagy, reduces senescence and ECM degradation	Only in animal/cell models; no human OA data	(127, 128)
	Liver-targeted drug delivery	Preclinical/technological platform	Silences pathogenic genes in liver to indirectly modulate OA	No OA-specific RCT data exist; conceptual proposal only	(129–131)
Kidney-Joint axis	Clearance of uremic toxins	Preclinical/CKD context only (no OA data)	Adsorbs uremic toxin precursors; may improve CKD-related bone metabolism	Evaluated for CKD outcomes, not for joint disease; no OA trials	(133, 143)
	Vitamin D supplementation	RCTs available with mixed/null results	Enhances chondrocyte autophagy, inhibits inflammation	trial showed no reduction in knee pain or cartilage volume loss; efficacy may depend on baseline status and supplementation timing	(142, 147, 185, 186)

FMT, Fecal microbiota transplantation; HPA, Hypothalamic-Pituitary-Adrenal axis; ECM, extracellular matrix; OA, osteoarthritis; CKD, chronic kidney disease; RCT, Randomized Controlled Trial.

### Evidence grading

To enhance transparency, we qualitatively graded the strength of evidence for each major pathway as described in the results sections: “strong evidence” (consistent findings from at least two independent human studies with mechanistic support from animal models), “moderate evidence” (primarily from preclinical models with some human correlative data), or “preliminary/emerging evidence” (single studies, or *in vitro* data without *in vivo* validation). Knowledge gaps and contradictory findings (e.g., negative Mendelian randomization results for gut microbiota) are explicitly discussed within each subsection.

## 2 Revisiting OA as a potential systemic disorder: perspectives on the gut-brain-liver-kidney axis

The understanding of OA may be undergoing a significant transformation. Traditionally characterized as a localized joint pathology, OA is now being re-evaluated for its potential links to extra-articular organs. In this review, we propose the “gut-brain-liver-kidney axis” as a conceptual framework to investigate whether and how these interconnected metabolic, inflammatory, and neuroendocrine networks could modulate the pathogenesis of OA. This axis plays a crucial role in modulating the pathogenesis of OA through interconnected metabolic, inflammatory, and neuroendocrine networks. The gastrointestinal tract is central to this process, as dysbiosis and barrier dysfunction can lead to systemic inflammation. Recent advancements have uncovered specific gut-joint signaling pathways, such as the C. bolteae–GUDCA–intestinal FXR–GLP-1 axis, which illustrate how gut-derived bile acids can remotely influence cartilage homeostasis (10). Bacterial extracellular vesicles have emerged as crucial nanoscale messengers, transporting microbial components into the joint and affecting local inflammation and cartilage integrity (11).

Beyond the gastrointestinal system, central sensitization within the brain-joint axis exacerbates chronic pain, frequently independent of structural damage (12). Disorders of lipid and iron metabolism originating in the liver are linked to the emerging phenomenon of chondrocyte ferroptosis (13). Renal dysfunction worsens OA by promoting the buildup of uremic toxins and disrupting vitamin D metabolism, both of which contribute to joint degeneration (9, 14). Taken together, these findings not only elucidate OA as a multi-system disorder but also reveal several novel treatment avenues targeting the joint. The subsequent sections will examine the specific roles of each organ system in the pathogenesis of OA and their therapeutic implications.

## 3 The gut-joint axis

### 3.1 Imbalanced intestinal flora compromises barrier integrity and promotes low-grade systemic inflammation

The gut microbiota consists of a wide variety of microorganisms, such as bacteria, archaea, and eukaryotes, which carry out vital physiological functions for the host. This complex community is crucial for modulating host immunity, maintaining and restoring the intestinal mucosal barrier, and preventing pathogen invasion (6). However, physiological functions that support the body can be negatively impacted by changes in microbial composition, a condition referred to as gut microbiota dysbiosis. While the number of clinical studies on this topic is relatively small and research methodologies differ—encompassing variations in sample sources, sequencing technologies, and taxonomic classifications—these discrepancies can influence the comparability of results. Nonetheless, many studies have established a correlation between shifts in the gut microbiota and the onset and progression of OA (15).

Research indicates that patients with osteoarthritis demonstrate a significant reduction in gut microbiota diversity and an imbalance in the proportions of specific microbial populations (16). Protective microbiota, such as Bifidobacterium, Bacteroides, Fecalibacterium, Roseburia, and other associated beneficial bacteria, are reduced (17). *Methanobacteriaceae* and *Desulfovibrionales* are associated with a reduced risk of OA (18). *Lactobacillus* (such as *L. casei Shirota, L. paracasei M5*) and *Bifidobacterium longum* showed significant increases after probiotic intervention, which were associated with the alleviation of OA symptoms (16). The increase in pathogenic microbiota, such as *Streptococcus* and *Enterococcus*, is positively correlated with OA pain and severity (17). The increase in Fusobacteriumand Ruminococcaceae is correlated with exacerbated inflammation in patients with OA and metabolic syndrome. Conversely, the decrease in Fecalibacterium may exacerbate disruption of the intestinal barrier (19); Clostridium was found to be upregulated in five studies and is associated with high-fat diet (HFD) and obesity-related OA models. A significant increase in the Firmicutes/Bacteroidetes (F/B) ratio indicates dysbiosis (16). The ratio of beneficial flora in OA has decreased, whereas the proportion of pathogenic flora has increased.

However, the research presents contradictory findings; some studies indicate a positive correlation between Lactobacillus and OA (20). Furthermore, Mendelian randomization studies did not find a direct association between OA and the gut microbiota (21). While the majority of studies we cite—including observational research documenting reduced protective bacteria, increased pathogenic species, and compromised intestinal barrier integrity, as well as animal experiments delineating the LPS–TLR4–NF-κB inflammatory pathway—consistently support an association between gut microbiota dysbiosis and osteoarthritis, a two-sample Mendelian randomization study failed to detect a causal link. However, this negative result must be interpreted with caution because of critical limitations: the gut microbiome GWAS included only approximately 3,300 individuals, yielding weak instrumental variables that severely limit statistical power to detect true effects; the exposure was defined as the overall gut microbiome rather than specific taxa, diluting any potential causal signals; and the outcome definition did not distinguish OA subtypes or inflammatory phenotypes. Therefore, the MR finding does not falsify the gut–joint axis hypothesis but rather reflects methodological constraints. Taken together, the available evidence firmly supports a correlational and mechanistic relationship, but causal inference in humans remains inconclusive, warranting larger-scale microbiota GWAS, refined exposure definitions, and further experimental validation. The implications of our findings suggest that larger-scale animal studies and clinical validations are required, along with a more comprehensive exploration of the underlying mechanisms. Typically, the microbiota alterations observed in OA patients involve an increase in pro-inflammatory species and a reduction in anti-inflammatory ones. This persistent dysbiosis of the gut microbiota can compromise both the structural integrity of the intestines and their barrier function, ultimately impacting the progression of OA (22).

The intestinal barrier is upheld by two key aspects: structure and function. The structure includes four layers: the intestinal lumen, alkaline phosphatase, the mucosal layer, tight junctions between epithelial cells, antimicrobial proteins from Paneth cells, and immunoglobulin A from immune cells in the lamina propria. The barrier maintains microbiota balance and prevents infections and inflammation. Certain strains of microbiota can influence the mucosal outer layer. Functionally, the intestine isolates harmful substances like bacteria and toxins, preventing their entry into other tissues, organs, and the bloodstream (23). Dysbiosis in the gut microbiota can impair the function of the gut barrier, resulting in increased intestinal permeability. This condition allows toxic bacterial metabolites to enter systemic circulation, which can contribute to low-grade systemic inflammation.

**Figure 1:**
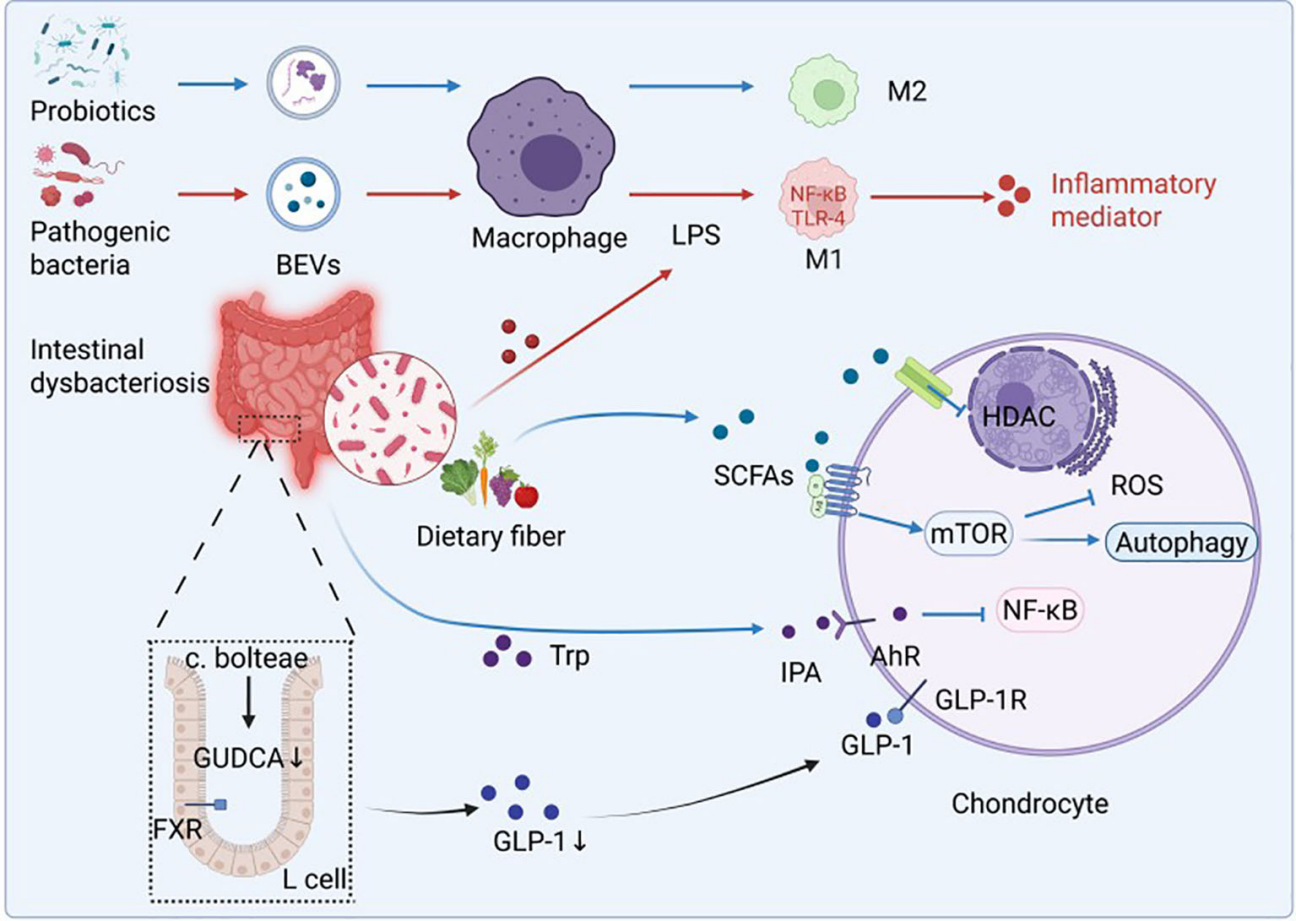
Schematic diagram illustrating how gut microbiota influences chondrocytes through multiple metabolic mechanisms. Probiotics and pathogenic bacteria regulate macrophage polarization (M1/M2) via BEVs; concurrently, gut microbiota dysbiosis induces LPS production, which promotes macrophage polarization toward the M1 phenotype and activates the NF-κB inflammatory pathway, thereby facilitating the secretion of inflammatory mediators. Dietary fiber is metabolized by gut microbiota to generate SCFAs, which inhibit HDAC, activate the mTOR signaling pathway, promote chondrocyte autophagy, and reduce the production of ROS. IPA, a metabolite of tryptophan, reduces the release of pro-inflammatory cytokines by inhibiting the NF-κB pathway. Dysregulation of the intestinal *Clostridium bolteae* population leads to decreased GUDCA levels, which in turn reduce GLP-1 secretion by L cells, ultimately accelerating OA progression. LPS, Lipopolysaccharide; TLR-4, Toll-like receptor 4; BEVs, Bacterial extracellular vesicles; SCFAs, short-chain fatty acids; HDAC, Histone Deacetylase; ROS, reactive oxygen species; Trp, Tryptophan; IPA, indole-3-propionic acid; AhR, aryl hydrocarbon receptor; GUDCA, Glycoursodeoxycholic acid; FXR, Farnesoid X receptor; GLP-1, glucagon-like peptide-1. The red arrows represent inflammatory pathways, the blue arrows represent anti-inflammatory pathways, and the black arrows represent the mapping of conventional mechanisms. **Figure 1** was created using BioRender. Created in BioRender.tao, S. (2026) https://BioRender.com/99j3y5o.

Furthermore, dysbiosis activates the innate immune system, causing an increase in pro-inflammatory factors that can negatively impact joint health (24). Once the gut microbiota’s dynamic equilibrium is disrupted, the integrity and functions of the intestinal barrier are compromised. The tight junctions between intestinal epithelial cells become impaired, allowing bacteria and lipopolysaccharides (LPS) in the intestinal lumen to enter the bloodstream through paracellular transport. LPS primarily originates from the outer membrane of Gram-negative bacteria, such as Bacteroides and Desulfovibrio. This process activates the NF-κB signaling pathway, transforming macrophages into M1-type macrophages, which interact with Toll-like receptor 4 and induce the production of pro-inflammatory factors, including IL-1β, tumor necrosis factor-alpha (TNF-α), matrix metalloproteinases (MMPs), and free radicals (25). This results in heightened intestinal permeability, which leads to increased serum levels of LPS and worsens disease progression (26, 27). A pathway-level schematic of these networks is presented in **Figure 1**, and their key molecular components are summarized in **Table 1**. By fueling systemic low-grade inflammation, gut dysbiosis contributes to OA pathogenesis. These findings challenge conventional understanding, reframing the disease and strengthening its theoretical basis as a metabolic inflammatory disorder.

Evidence level: Preliminary to moderate. The conclusions regarding gut microbiota compositional differences between OA patients and healthy controls rest primarily on cross-sectional and case-control studies, providing observational, associative evidence. Although some microbial alterations have been replicated in animal models, a causal relationship remains to be established. Mendelian randomization studies have not identified direct causal links. Therefore, the current evidence is graded as preliminary/emerging (human observational studies plus preclinical models). Larger prospective cohorts and mechanistic validation are warranted. Despite its significant potential, research in this field is still in its early stages. Therefore, it is essential to explore the molecular mechanisms of the gut-joint axis to identify specific microbial signatures and functions associated with OA.

### 3.2 Short-chain fatty acids and tryptophan metabolites exert anti-inflammatory and chondroprotective effects

The role of gut dysbiosis in the pathogenesis of OA is influenced by a range of metabolites derived from the gut, particularly short-chain fatty acids (SCFAs) and tryptophan catabolites. The regulatory mechanisms through which these metabolites impact joint health by modulating inflammatory responses and the immune system are illustrated in **Figure 1**. SCFAs, which are predominantly produced when gut microbiota, such as Bacteroides and Roseburia, ferment dietary fibers, possess significant anti-inflammatory properties. These fatty acids can modify immune cell functions either by inhibiting histone deacetylases or by activating G protein-coupled receptors, including GPR43 (28, 29). The mTOR signaling pathway plays dual roles in chondrocyte metabolism and OA progression. SCFAs promote anabolic metabolism by activating mTOR, while concurrently inhibiting the production of reactive oxygen species (ROS). This inhibition mitigates oxidative stress-related cartilage damage. Research indicates that butyrate enhances chondrocyte autophagy, promoting the clearance of damaged mitochondria and thereby delaying the progression of OA (30, 31). SCFAs primarily function to attenuate joint inflammation and prevent cartilage breakdown.

Tryptophan, an essential aromatic amino acid, has metabolites largely produced by the gut microbiome, particularly by Lactobacillus species, which convert tryptophan into aryl hydrocarbon receptor (AhR) ligands. These ligands activate the AhR, resulting in the downregulation of IL-17 levels, which are associated with joint degeneration and tissue aging associated with OA (32). Additionally, tryptophan metabolites, such as indole-3-sulfate, indole-3-propionic acid, and indole-3-aldehyde, can activate AhR signaling in astrocytes, inhibit inflammation in the central nervous system, and may be involved in neuropathic pain associated with OA (33, 34). Notably, indole-3-propionic acid (IPA) demonstrates anti-inflammatory properties across various diseases. IPA significantly suppresses the expression of inflammatory factors, including nitric oxide, prostaglandin E2 (PGE2), TNF-α, interleukin-6 (IL-6), inducible nitric oxide synthase (iNOS), and cyclooxygenase-2 (COX-2), as well as matrix-degrading enzymes induced by IL-1β, such as MMP-3, MMP-13, and ADAMTS-5 (35–37). Furthermore, IPA upregulates anabolic markers, including aggrecan and collagen-II, while inhibiting the NF-κB pathway. Beyond lowering serum levels of inflammatory cytokines, IPA—a tryptophan metabolite—also modulates cartilage degradation and synovitis *in vivo*. These metabolites may further influence central nervous system inflammation and neuropathic pain, thereby alleviating joint degeneration and pain, ultimately serving a protective role in OA progression (38).

SCFAs create a systemic anti-inflammatory state by strengthening the gut barrier and regulating immunity, providing both indirect joint protection and potential direct benefits to chondrocytes. The metabolism of tryptophan dictates the progression of inflammation. In summary, these two classes of metabolites are key mediators in the gut-joint axis, and their critical information and signaling pathways are summarized in **Table 1**. Understanding their roles informs therapeutic strategies focused on gut-centric interventions in OA.

Evidence level: Preliminary (preclinical mechanistic studies). The regulatory effects of SCFAs and tryptophan metabolites (e.g., IPA) on chondrocytes and inflammation have been demonstrated primarily *in vitro* (e.g., in IL-1β-stimulated chondrocytes) and in rodent OA models. Evidence that IPA suppresses inflammation via the AhR/NF-κB pathway likewise derives largely from cellular and animal studies. Although observational studies have documented altered levels of certain metabolites in the serum or feces of OA patients, direct causal evidence confirming their protective actions within human joints remains at the preclinical stage. Thus, the evidence is graded as preliminary/emerging.

### 3.3 The GUDCA-FXR-GLP-1 axis links bile acid metabolism to cartilage homeostasis

Bile acids are potent metabolic and immune signaling molecules. Synthesized from cholesterol in the liver, they are transported to the intestine and further metabolized by the gut microbiota. The composition of the bile acid pool is shaped by the combined enzymatic activities of the host and the microbiota, giving rise to substantial compositional diversity that depends, in part, on differences in gut bacterial species. Disruption of bile acid signaling caused by gut microbiota dysbiosis or dysregulated microbiota–host interactions has been implicated in the onset and progression of metabolic disorders (39). The primary mechanism through which gut microbiota and bile acid metabolism contribute to joint homeostasis is illustrated in **Figure 1**. Research has shown that plasma concentrations of glycoursodeoxycholic acid (GUDCA) are significantly inversely correlated with both the occurrence and severity of OA. This observation was further corroborated by the independently conducted Xiangya Walking Study (10). GUDCA alleviates OA progression in mice by selectively inhibiting the intestinal farnesoid X receptor (FXR), a known negative regulator of glucagon-like peptide-1 (GLP-1) secretion (40). Reduced expression of the FXR in intestinal stem cells results in the expansion of L-cells, which in turn boosts GLP-1 secretion and elevates systemic GLP-1 levels. This phenomenon is clinically significant, as circulating GLP-1 levels are notably lower in OA patients, and serum GLP-1 levels show a positive correlation with synovial fluid levels. Furthermore, *in vivo* studies have confirmed that intra-articular injections of GLP-1 agonists can alleviate inflammatory conditions (41, 42). Epidemiological data also support an association between GLP-1 receptor agonist use and a reduced risk of OA progression, strongly bolstered by a recent randomized controlled trial demonstrating the efficacy of semaglutide in knee OA patients (43).

Metagenomic sequencing demonstrated a reduced relative abundance of Clostridium bolteae in the gut microbiota of OA patients. Multi-omics analyses established a positive correlation between the abundance of C. bolteae and plasma GUDCA levels (10). Supplementation with C. bolteae in mice increased levels of the GUDCA precursor ursodeoxycholic acid (UDCA) and GLP-1, thereby slowing OA progression. Importantly, the widely used and well-tolerated drug UDCA was found to inhibit intestinal FXR activity, promote L-cell differentiation and GLP-1 secretion, and delay OA progression in mice. Clinical data further indicated that oral UDCA is associated with a reduced risk of OA progression, suggesting its potential for repurposing in OA treatment (10).

TGR5, also known as GPBAR1, is a G protein-coupled receptor that responds to various bile acids, such as lithocholic acid (LCA), deoxycholic acid (DCA), and chenodeoxycholic acid (CDCA). Expressed in a wide range of tissues, including bone, intestine, and immune cells, TGR5 critically modulates bone metabolism and inflammatory responses (44, 45).

TGR5 contributes to the synthesis of cartilage extracellular matrix (ECM). Activation of TGR5 stimulates the secretion of GLP-1 from intestinal L cells; GLP-1 subsequently acts on chondrocytes through its receptor (GLP-1R) to promote the production of ECM components such as aggrecan (ACAN) and type II collagen (COL2A1), while suppressing the expression of matrix-degrading enzymes MMP13 and ADAMTS5, thereby attenuating cartilage degradation. In parallel, bile acids such as UDCA and GUDCA indirectly enhance GLP-1 secretion by inhibiting FXR and activating TGR5, which in turn reinforces cartilage matrix synthesis and ameliorates OA symptoms (10, 46, 47). TGR5 attenuates chondrocyte inflammation and apoptosis. Activation of TGR5 suppresses the NF-κB signaling pathway, thereby downregulating the expression of MMPs and ADAMTS induced by pro-inflammatory cytokines such as IL-1β and TNF-α, and reducing ECM degradation. Additionally, TGR5 modulates osteoclast differentiation through the AMPK signaling pathway, which diminishes bone resorption and indirectly preserves the structural integrity of subchondral bone (47–49). TGR5 exerts a dual regulatory role. Although TGR5 activation has been shown to be protective in the majority of studies, certain bile acids, such as deoxycholic acid (DCA), upregulate tryptophan hydroxylase 1 (TPH1) expression via TGR5, thereby promoting intestinal serotonin (5-HT) release. This may enhance bone resorption, suggesting that the effects of TGR5 are both metabolite-specific and concentration-dependent (50). TGR5 agonists, such as INT-777, and natural bile acids including UDCA and GUDCA are regarded as potential therapeutic strategies for OA, capable of improving cartilage metabolism through modulation of the gut–joint axis. Further investigation is needed to delineate the downstream signaling network of TGR5 in chondrocytes and to elucidate its distinct roles across different stages of OA (10, 51).

This study reveals a novel mechanism of action, outlined in **Table 1**. Evidence level: Moderate (human observational plus RCT evidence for GLP-1 receptor agonists; some mechanisms remain preclinical). The core mechanism of the GUDCA–FXR–GLP-1 axis (FXR-mediated regulation of GLP-1) has been established primarily in mouse and cell models, representing preclinical evidence. Human observational cohort studies support associations between GUDCA, C. bolteae, and OA, and RCTs have demonstrated clinical benefits of GLP-1 receptor agonists in OA patients. By contrast, the protective role of TGR5 remains at the preclinical stage and has yet to be validated in humans. Overall, the evidence is graded as moderate, but additional human mechanistic studies (e.g., intestinal biopsy, FXR-targeted interventions) are required to confirm translational potential.

### 3.4 Bacterial extracellular vesicles act as nanoscale messengers in gut-joint communication

Nanoparticle-sized, lipid membrane-enclosed particles secreted by both Gram-negative (G^-^) and Gram-positive (G^+^) bacteria are referred to as bacterial extracellular vesicles (BEVs). BEVs play a crucial role in the regulation of joint function within the gut microbiota, with their regulatory pathway outlined in **Figure 1**. These phospholipid bilayer vesicles, measuring 20–400 nm in diameter, facilitate communication between bacteria and the host, allowing for the transport and regulation of various bacterial bioactive components, including nucleic acids, LPS, SCFAs, and toxins (11). The functional diversity, irreproducibility, low toxicity, high biocompatibility, ease of modification, and scalability of BEVs give them significant potential in biomedical applications. The mechanisms of BEV formation and the characteristics of their cargo vary between G+ and G- bacteria, reflecting their distinct cellular structures and physiological functions. BEVs’ function depends on the source strain’s identity and status: vesicles from a dysbiotic microbiome or pathogenic bacteria can exacerbate OA, while those from probiotics may provide therapeutic benefits. A summary of these divergent roles is provided in **Table 1**. Gram-negative bacteria can produce outer membrane vesicles (OMVs) through budding from the outer membrane, as well as generate outer inner membrane vesicles (OIMVs) and extra outer membrane vesicles (EOMVs) via explosive cell lysis (52); G+ bacteria produce Cell membrane vesicles (CMV) through the process of bubble cell death (53).

BEVs exhibit a diverse composition, while OMVs are predominantly composed of LPS, outer membrane proteins, and peptidoglycan. Conversely, OIMVs or EOMVs primarily contain cytoplasmic materials such as DNA and RNA, along with phage components. CMVs mainly consist of cytoplasmic proteins, toxins, and genetic material. When the intestinal barrier is compromised, pathogenic bacterial BEVs, such as those derived from Escherichia coli, degrade tight junction proteins like ZO-1 and occludin. This degradation increases intestinal permeability and facilitates the entry of endotoxins into the bloodstream, triggering systemic chronic low-grade inflammation, which subsequently results in joint damage (54). Probiotic-derived extracellular vesicles, such as Nissle 1917, have been shown to enhance barrier integrity and possess anti-inflammatory properties (55).

Additionally, BEVs play a significant role in immune-inflammatory responses. In pro-inflammatory pathways, BEVs activate the TLR/NOD receptors of joint macrophages through LPS/PG, leading to the release of inflammatory factors like TNF-α and IL-6, which accelerates cartilage catabolism (22, 26, 56); In the anti-inflammatory pathway, probiotic-derived extracellular vesicles from Lactobacillus johnsonii promote macrophage polarization toward the M2 phenotype, suppressing inflammation and protecting cartilage (57); The genetic material, whether DNA or RNA, of BEVs is delivered to articular cartilage through OIMV or EOMV, thereby impacting the gene expression of chondrocytes. This process may occur via neovascular invasion or through cartilage fissures, leading to a local microecological imbalance that influences the progression of OA (58, 59). BEVs, which are nanoscale phospholipid bilayer vesicles secreted by gut microbiota, have the capacity to transport diverse bioactive molecules and regulate host physiological and pathological processes remotely. They are emerging as a novel class of messengers that connect the gut and joints.

Evidence level: Preliminary (almost exclusively from preclinical models). Current understanding of BEV structure, composition, and their effects on the intestinal barrier and immune cells derives largely from bacterial culture, *in vitro* cell models, and mouse models. Evidence that Lactobacillus johnsonii-derived extracellular vesicles delay OA progression is likewise based on animal experiments. Direct detection, abundance changes, and pathogenic roles of BEVs within human OA joints remain exceedingly understudied. Thus, the evidence is graded as preliminary/emerging, and validation using human samples is required.

### 3.5 Targeting the gut-joint axis: probiotics, fecal microbiota transplantation, prebiotics and bioactive compounds

#### 3.5.1 Probiotics

Probiotics are defined as live microorganisms that, when administered in adequate amounts, confer health benefits to the host. By modulating the composition of gut microbiota, probiotics may reduce systemic inflammation and, consequently, alleviate OA symptoms, such as joint pain and functional impairment. The underlying mechanisms may involve the suppression of inflammatory pathways and the enhancement of intestinal barrier integrity, as summarized in **Table 2**. Both preclinical studies utilizing animal models and clinical trials examining specific probiotic strains for the treatment of OA have demonstrated promising therapeutic outcomes (60).

Preclinical evidence from animal models indicates that Lactobacillus casei can alleviate pain by reducing synovial levels of COX-2, TNF-α, and MMPs, while also upregulating TIMP-1 and type II collagen (31). Lactobacillus acidophilus has been shown to suppress the expression of TRPV1 and CGRP in dorsal root ganglia, enhance the abundance of butyrate-producing bacteria, particularly within the family Lachnospiraceae, alleviate joint pain, reduce cartilage degradation, and decrease synovial inflammation. This multifaceted mechanism leads to a reduced IL-1β/TNF-α ratio (31). L. rhamnosus promotes GABA release and PPAR-γ expression, reduces MCP-1/CCR2, and alleviates pain and cartilage damage (61). Clostridium butyricum inhibits inflammatory factors such as IL-1β and TNF-α by secreting butyrate, thereby improving osteophyte formation and cartilage degeneration, reducing OA severity, and increasing load-bearing capacity. Additionally, Streptococcus thermophilus has the ability to produce hyaluronic acid, which helps alleviate joint swelling and synovial inflammation while also reducing chondrocyte apoptosis (60).

A randomized controlled trial was conducted using L. casei Shirota. A total of 461 patients with knee OA were enrolled over six months and received a daily dose of 12 × 10*9 CFU. The final observations revealed a decrease in Visual Analog Scale (VAS) scores, Western Ontario and McMaster Universities OA Index (WOMAC) scores, and serum high-sensitivity C-reactive protein (hs-CRP) levels. Furthermore, a robust linear correlation was identified among the three variables: hs-CRP levels, WOMAC scores, and VAS scores (62). Another study systematically reviewed randomized controlled trials on probiotic treatment for OA-related pain and inflammation, assessing self-reported pain, stiffness, disability, and serum hs-CRP levels. Three studies involving 501 participants were eligible for qualitative synthesis and meta-analysis. All measured outcomes for Lactobacillus casei Shirota, except stiffness, demonstrated significant symptom alleviation (63). Probiotics, especially those of the Lactobacillus genus, have shown promise in mitigating pain and inflammation associated with OA by modulating the gut-joint axis. This indicates that they could serve as a valuable adjunctive treatment strategy for OA.

However, the effectiveness of probiotics is greatly dependent on the specific strains utilized. Future research should prioritize large-sample, long-term randomized controlled trials that incorporate multi-omics technologies to establish personalized treatment protocols. Additionally, it is crucial to conduct further studies to explore the role of probiotics in human OA more comprehensively (64). Probiotics may affect OA pain management through various mechanisms, including the modulation of gut microbiota, suppression of systemic inflammation, and regulation of pain pathways. While current clinical evidence remains limited, preliminary findings from both animal and clinical studies show promise. To establish their effectiveness and support their incorporation into a multimodal treatment approach, future high-quality research is essential.

Evidence level: Probiotics – moderate; FMT – preliminary; prebiotics – preliminary to moderate; bioactive compounds – preliminary. Probiotics: RCTs (e.g., L. casei Shirota) show symptomatic and inflammatory benefits, but sample sizes are small and effects are strain-dependent. Evidence level: moderate. FMT: Only tested in animal models for the gut-joint axis; no RCT in human OA. Evidence level: preliminary. Prebiotics: Small RCTs suggest improvements in pain and hs-CRP, but larger studies with standardized formulations are needed. Evidence level: preliminary to moderate. Bioactive compounds: Mostly preclinical; a few RCTs (e.g., curcumin) show symptomatic relief, but mechanisms and clinical efficacy require confirmation. Evidence level: preliminary.

#### 3.5.2 Fecal microbiota transplantation

Fecal microbiota transplantation (FMT) is a therapeutic procedure in which functional microbial communities from a healthy donor’s feces are transferred into a recipient’s gastrointestinal tract. This intervention aims to restore the gut microbiota and address both intestinal and extraintestinal disorders, as detailed in **Table 2**. FMT marks a significant advancement in the field of microbiota transplantation technology (65). FMT has been proven highly effective in treating recurrent Clostridium difficile infections (66).

An interesting attempt to explore the feasibility of FMT was performed by Huang et al. in mice (19). Fecal samples were collected from three distinct groups: a healthy human control group, a knee OA group without metabolic syndrome, and a knee OA group with metabolic syndrome. The gut microbiota from patients with metabolic syndrome (MetS) exacerbated OA induced in germ-free mice following ankle ligament injury following FMT. This finding aligns with the authors’ proposed ‘second hit’ theory, wherein dysbiosis of the gut microbiota represents the first hit. Specifically, microbial communities associated with MetS compromise the intestinal barrier by reducing the expression of tight junction proteins such as ZO-1 and occludin. This reduction increases intestinal permeability, allowing LPS to enter the bloodstream and trigger systemic low-grade inflammation. The second hit originates from joint injury; surgical intervention releases damage-associated molecular patterns that synergistically activate inflammatory pathways, accelerating OA progression. Collectively, these two hits contribute to the worsening of OA. This study underscores the significance of FMT in understanding OA pathogenesis and provides hope for manipulating the gut microbiota as a strategy for managing OA.

However, due to the limited sample size, further comprehensive studies are warranted. Future strategies may involve modulating specific bacterial genera, such as suppressing Fusobacterium while supplementing Ruminococcaceae. FMT presents a dual-edged sword for OA management: microbiota from metabolically abnormal donors can exacerbate joint damage by disrupting the intestinal barrier and triggering systemic inflammation, particularly post-transplantation. Conversely, microbiota from healthy donors have the potential to reshape the immune microenvironment, establish a more resilient microbial ecosystem, promote immune cell differentiation, suppress inflammation, and repair the intestinal barrier, thus paving the way for innovative treatment approaches for OA (67). Future research should focus on elucidating the functions of key strains and their subtypes, refining donor selection strategies, and advancing precision microbiota interventions for therapeutic purposes (68).

#### 3.5.3 Prebiotics

Beyond live microorganisms, prebiotics—defined as non-digestible substrates that selectively stimulate the growth or activity of beneficial gut microbes—have emerged as promising adjunctive strategies for OA management (69). Unlike probiotics, prebiotics such as inulin, fructo-oligosaccharides (FOS), and galacto-oligosaccharides (GOS) modulate the gut ecosystem by enriching native populations of Bifidobacteria and Lactobacilli, thereby avoiding potential risks associated with exogenous bacterial administration.

Several RCTs have provided preliminary evidence for prebiotic intervention in OA. A 6-month RCT demonstrated that daily supplementation with oligofructose-enriched inulin significantly reduced serum levels of high-sensitivity C-reactive protein (hs-CRP) and improved the WOMAC pain scores in patients with knee OA, compared with placebo (70). Mechanistically, prebiotic fermentation yields SCFAs in the gut lumen, which not only reinforce intestinal barrier integrity by upregulating tight junction proteins (e.g., claudin-1, occludin) but also enter the systemic circulation to directly modulate chondrocyte autophagy via the GPR43-mTOR axis (29, 30). These findings suggest that prebiotics could serve as a safe, low-cost dietary intervention to suppress the low-grade systemic inflammation that fuels OA progression. However, larger-scale RCTs with standardized formulations and longer follow-up are warranted to confirm their disease-modifying potential.

#### 3.5.4 Bioactive compounds

A wide array of naturally occurring bioactive compounds has demonstrated therapeutic potential in OA by targeting the gut-joint axis and systemic inflammation, independent of conventional probiotic or prebiotic effects (71). These phytochemicals and nutraceuticals exert multi-target anti-inflammatory and chondroprotective actions, often by modulating gut microbiota composition or directly inhibiting catabolic signaling pathways in articular tissues.

Polyphenols and flavonoids are among the most extensively studied classes. Resveratrol suppresses the NF-κB pathway in chondrocytes and simultaneously enriches gut Akkermansia muciniphila, a beneficial bacterium associated with enhanced intestinal barrier function (72, 73). Similarly, curcumin (from turmeric) and its more bioavailable formulations have produced consistent symptomatic relief in multiple RCTs, with meta-analyses indicating effects comparable to nonsteroidal anti-inflammatory drugs (NSAIDs) but with fewer gastrointestinal adverse events (74). The anthocyanins from berries and other flavonoids like quercetin and epigallocatechin-3-gallate (EGCG) have also shown anti-ferroptotic and anti-apoptotic effects in pre-clinical OA models by scavenging reactive oxygen species and upregulating glutathione peroxidase 4 (GPX4) (75, 76).

Terpenoids and alkaloids represent another promising category. Celastrol, a triterpenoid extracted from Tripterygium wilfordii, has been found to alleviate cartilage degradation in animal models by inhibiting the NLRP3 inflammasome and promoting mitophagy (77). Although clinical application of some bioactive compounds has been limited by poor oral bioavailability, emerging formulation strategies—including nanoparticle encapsulation and phospholipid complexes—are overcoming these obstacles. Future studies should prioritize head-to-head RCTs comparing specific bioactive compounds with standard OA therapies, along with rigorous mechanistic dissection of their microbiota-dependent versus direct actions on articular tissues.

## 4 The brain-joint axis

### 4.1 Central sensitization drives maladaptive pain and creates a vicious cycle of functional impairment

The pain associated with OA stems predominantly arises from nociceptive stimulation resulting from injury to the joint cartilage. However, ongoing research has demonstrated that the pain experienced by patients with OA does not consistently correlate with the extent of structural damage observed in their joints (7). Research has found that some patients with significant joint damage report minimal pain, while others with minor joint alterations suffer from severe chronic pain (78). This paradoxical phenomenon demonstrates that central sensitization (CS) plays a crucial regulatory role in chronic pain associated with OA. CS is characterized by an abnormally heightened responsiveness of the central nervous system (primarily the spinal cord and brain) to sensory inputs, especially nociceptive stimuli. Its core features include hyperalgesia, pain spread, pain persistence, and the development of allodynia (79). CS and peripheral nociceptive mechanisms are not distinct entities; rather, they are components of a closely interconnected regulatory network. This system enables peripheral inflammatory signals to enhance central sensitization, which in turn modulates peripheral inflammation through descending control pathways (80).

Before detailing the mechanisms, it is crucial to distinguish the role of the brain in OA pathogenesis. Unlike the gut, liver, or kidney, which influence OA primarily through systemic metabolic or inflammatory pathways that directly contribute to cartilage degradation and synovitis, the brain’s role is twofold. First, it is the center for pain perception and modulation, a process known as central sensitization (CS). Second, through neuroendocrine pathways, it can indirectly impact joint health. This section will first focus on CS as a key mechanism of pain amplification, clarifying that it does not directly erode cartilage but fundamentally alters the patient’s pain experience and subsequent behaviors, thereby creating a vicious cycle that secondarily exacerbates structural damage.

The presence of central sensitization has been confirmed through various methods (12). Existing research has shown that chronic pain can lead to CS, which alters various brain functions. The initial drive and ongoing stimulation are essential, as persistent inflammatory factors, tissue-damage products, and mechanical stresses continuously activate peripheral nociceptors in OA joints. This sustained nociceptive input initiates and maintains CS, resulting in abnormalities in the nervous system, particularly in the spinal cord. The mechanism of CS primarily stems from ongoing peripheral nociceptive input, including inflammatory mediators in the joints (e. g. IL-1β, IL-6, TNF-α, NGF, PGE2), mechanical damage, and pathological changes, which continuously stimulate chronic pain through primary afferent nerve fibers (C fibers and Aδ fibers) that transmit signals to the dorsal horn of the spinal cord (81). Over time, dorsal horn neurons in the spinal cord experience sensitization, resulting in heightened excitability to incoming signals and an increased responsiveness to repeated stimuli. The underlying mechanisms of this facilitation are outlined in **Table 1**. This sensitization causes an expansion of the neuronal receptive field, enabling neurons to respond not only to stimulation and pain from adjacent joints but also to stimuli affecting more distant body regions, such as the contralateral joint or non-articular areas.

Furthermore, the pain threshold is lowered, allowing non-noxious stimuli to provoke pain sensations. The ongoing transmission of synaptic signals may lead to long-term potentiation, a process marked by a sustained increase in the efficiency of synaptic transmission, which is thought to serve as the cellular foundation for CS and chronic pain memory (78, 81). In addition, glial cells, especially spinal microglia and astrocytes, become abnormally activated by peripheral inflammatory signals. This activation results in the release of pro-inflammatory cytokines and other nociceptive substances that enhance the hyperexcitability of primary sensory neurons, thereby contributing to OA pain. The abnormal excitability of neurons is frequently linked to microglial hyperactivity in the spinal dorsal horn. Under normal physiological conditions, microglia in the spinal cord remain in a resting state, characterized by a highly branched morphology that allows for continuous monitoring of the microenvironment, functioning like sentinels.

However, persistent and intense peripheral nociceptive signals stemming from inflammatory factors in the joints activate microglia within the spinal dorsal horn. Upon activation, microglia undergo morphological transformations, shifting from a ‘branch-like’ form to an ‘amoeboid’ shape, which facilitates their migration and the release of various substances. Laboratory experiments have shown a considerable upregulation of molecular markers such as Iba1 (ionized calcium-binding adapter molecule 1) and CD11b (82). When neurons are strongly and persistently activated, they release CX3CL1, a key chemokine produced by neurons. CX3CL1 binds to the receptor CX3CR1, which is specifically expressed on microglia, serving as one of the most critical signals for activating spinal microglia (83). Microglia are activated via the p38/MAPK signaling pathway, resulting in the synthesis and release of various pro-inflammatory cytokines (82).

Increasing presynaptic glutamate release enhances pain signals by upregulating the function and expression of postsynaptic AMPA and NMDA receptors (84). Microglia in the spinal cord play a vital role in mediating and amplifying central sensitization associated with chronic pain related to OA. Their activation occurs in response to neuronal activity through the CX3CL1/CX3CR1 axis, which initiates the release of pro-inflammatory cytokines. This cascade of events induces functional remodeling of spinal neuronal circuits, ultimately resulting in CS (85). Once central sensitization is established in patients with OA, chronic pain and CS may influence the progression of OA through both passive and active mechanisms.

Throughout the pathological process, these factors can modulate multiple pathways in OA, potentially creating a vicious cycle. Firstly, prolonged chronic pain and abnormal pain sensitivity may lead OA patients to consciously or unconsciously limit their natural joint activity, actively decreasing joint movement. This reduction in activity results in varying degrees of disuse atrophy in both the joints and surrounding muscles. When muscle atrophy and weakness occur, the muscles around the joints—particularly the quadriceps—play a vital role as stabilizers and shock absorbers (86). Muscle atrophy and weakness directly undermine joint stability, increase abnormal loads, and accelerate further cartilage wear (87). This can result in joint stiffness and reduced mobility, which directly impairs the circulation of synovial fluid and the supply of nutrients, ultimately hindering repair processes and exacerbating arthritis. When individuals experience pain, they often subconsciously avoid engaging the muscles surrounding the affected area, instinctively adopting postures that offer comfort and protection. This behavioral adjustment changes the way stress is distributed across the joints, leading to abnormal local stress concentrations that accelerate cartilage degeneration and induce atypical bone remodeling, such as the formation of osteophytes (88).

**Figure 2:**
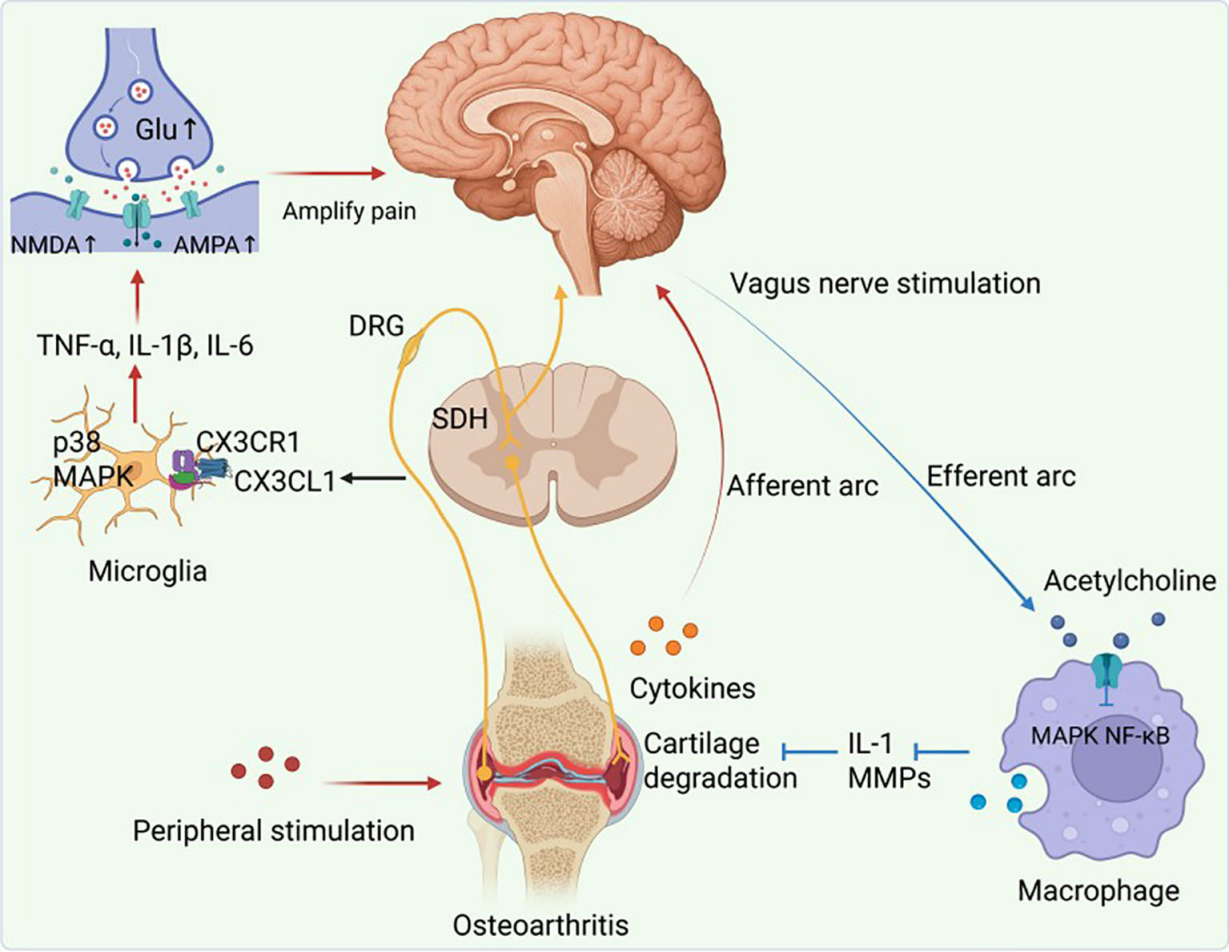
Schematic diagram showing the effects of central sensitization and neuroendocrine regulation mechanisms on joints. Sustained peripheral stimuli are transmitted to the central nervous system via the dorsal root ganglia. Within this pathway, neurons secrete the chemokine CX3CL1, which activates microglia in the dorsal horn of the spinal cord. These microglia are subsequently activated through the p38/MAPK signaling pathway, leading to the synthesis and release of numerous pro-inflammatory cytokines. Concurrently, increased presynaptic glutamate release and upregulation of AMPA and NMDA receptors amplify pain signaling. Local pro-inflammatory responses induced by these inflammatory factors can activate the parasympathetic nervous system through the afferent arc. The efferent arc then activates the vagus nerve via the cholinergic anti-inflammatory pathway, promoting acetylcholine release. Acetylcholine binds to α7nAChR on the surface of macrophages, and activation of α7nAChR inhibits the MAPK/NF-κB pathway, reduces pro-inflammatory cytokine release, and thereby alleviates cartilage degradation. TNF-α, Tumor Necrosis Factor-α; IL-β, Interleukin-β; IL-6, Interleukin-6; IL-1, Interleukin-1; MMPs, Matrix metalloproteinases; NGF, Nerve growth factor; PGE2, ProstaglandinE2; DRG, Dorsal root ganglion; SDH, spinal dorsal horn; CX3CL1, Chemokine (C-X3-C motif) ligand 1; CX3CR1, Chemokine (C-X3-C Motif) Receptor 1; NMDA, N-Methyl aspartic acid; AMPA, α-amino-3-hydroxy-5-methyl-4-isoxazole-propionic acid;α7nAChR, α7 nicotinic acetylcholine receptor. Red arrows indicate inflammatory pathways, blue arrows indicate anti-inflammatory pathways, yellow arrows indicate neural pathways, and black arrows indicate the depiction of conventional mechanisms. **Figure 2** was created using BioRender. Created in BioRender. tao, S. (2026) https://BioRender.com/xvyi4ty.

Central sensitization is a key neurobiological mechanism that significantly influences the onset and persistence of chronic pain in OA. This mechanism results in a complex pain profile marked by widespread pain distribution, hyperalgesia, and a disconnect between pain intensity and the degree of structural joint damage. The regulatory processes that govern central sensitization and hyperalgesia are illustrated in **Figure 2**. More importantly, chronic pain and the associated state of CS are not simply passive results of OA; instead they actively shape and enhance the overall disease progression through various mechanisms, including reduced physical activity, altered biomechanics, and psychosocial factors. This interaction fosters a detrimental cycle of “pain - functional impairment - exacerbation of structural damage - worsening pain.

Evidence level: Moderate (human functional evidence confirms the phenomenon, but molecular mechanisms derive from preclinical models). The core mechanisms of central sensitization (spinal microglial activation, p38 MAPK signaling, CX3CL1/CX3CR1 axis, NMDA receptor upregulation) have been delineated primarily in rodent pain models and ex vivo spinal cord slices/cell cultures. Human evidence from brainstem fMRI, PainDETECT questionnaire, and quantitative sensory testing confirms the presence of central sensitization in OA patients. However, direct evidence for these specific molecular pathways in the human CNS remains scarce. Thus, the evidence is graded as moderate (human functional data plus preclinical mechanisms).

### 4.2 Neuroendocrine dysfunction impairs endogenous anti-inflammatory capacity and joint homeostasis

The neuroendocrine system serves as a crucial link between brain stress responses, emotional states, metabolic activity, and the joint microenvironment. As summarized in **Table 1**, it regulates these processes through multiple pathways, including the hypothalamic-pituitary-adrenal (HPA) axis, the autonomic nervous system, and various neurotransmitters and neuropeptides. In patients with OA, prolonged chronic pain and a persistent state of stress can lead to dysregulation of the HPA axis, resulting in disturbances in glucocorticoid release, which subsequently affects joint inflammation and cartilage metabolism. Initially, there is hyperactivity of the HPA axis; however, this may later progress to exhaustion. Throughout this process, the rhythmicity and anti-inflammatory efficacy of glucocorticoids are compromised, leading to alterations in cortisol rhythms (89, 90). Dysregulation of the HPA axis diminishes the inhibitory effect of endogenous glucocorticoids on systemic low-grade inflammation. Furthermore, the imbalance of endogenous glucocorticoids negatively affects the maintenance of joint homeostasis (91). The ongoing low-grade inflammation observed in OA necessitates the modulation of a properly functioning HPA axis, which plays a crucial role as a counter-regulatory mechanism. When this axis becomes dysregulated—manifesting as a diminished stress response or disruption of its natural rhythmicity—it can result in an inability to control inflammation, thereby worsening the course of the disease.

The parasympathetic nervous system, an integral part of the autonomic nervous system, functions as a key regulatory link between the brain and the immune system. It significantly influences systemic inflammatory processes, demonstrating its vital role in the interplay between neural and immune functions (92). Local pro-inflammatory processes, often triggered by chronic pain or pathogens, can activate the autonomic nervous system, leading to various immunoregulatory mechanisms. The parasympathetic fibers play a crucial role in maintaining joint homeostasis and regulating local inflammation. Specifically, the activation of the vagus nerve through the cholinergic anti-inflammatory pathway facilitates the release of acetylcholine, which interacts with α7 nicotinic acetylcholine receptors on macrophages. This pathway’s potent anti-inflammatory effects, as demonstrated in other rheumatic diseases, are illustrated in **Figure 2**. Within the joint environment, the α7 nicotinic acetylcholine receptor is predominantly expressed on resident cells. When activated, this receptor inhibits the IL-1-induced MAPK/NF-κB signaling pathway, thereby reducing cartilage degradation (90). Data indicate that the vagus nerve is involved in joint homeostasis and the pathogenesis of OA, reducing the release of pro-inflammatory factors (93).

Neurotransmitters and neuropeptides play crucial roles in regulating pain and inflammation in OA. Notably, glutamate, the primary excitatory neurotransmitter within the somatosensory pain pathway, acts as a significant mediator of central sensitization. Increased concentrations of glutamate in the spinal cord and brain areas involved in pain perception are linked to the persistent pain associated with OA (94). After decades of research, it has become clear that OA is not merely a condition of “wear and tear.” Instead, OA is recognized as a dynamic disease affecting the entire joint, governed by both local and central nervous system mechanisms.

Evidence level: Moderate (human endocrine phenotypes plus preclinical mechanistic data). Human observational studies (e.g., cortisol rhythm, dexamethasone suppression tests) provide associative evidence linking HPA axis dysfunction to OA. Support for the vagus nerve–α7nAChR anti-inflammatory pathway comes primarily from animal arthritis models and *in vitro* macrophage experiments, with limited direct validation in OA patients. Thus, the evidence is graded as moderate, and confirmation requires human intervention studies (e.g., vagus nerve stimulation).

### 4.3 Multimodal therapies targeting central sensitization and neuroendocrine dysfunction

A comprehensive understanding of CS and neuroendocrine regulation in chronic pain associated with OA is essential for elucidating their core regulatory roles and potential impacts on disease progression. This understanding offers valuable guidance for the development of novel therapeutic strategies and possesses considerable clinical significance. Utilizing multidimensional assessment tools, such as the Central Sensitization Inventory, pain distribution maps, clinical assessments, quantitative sensory testing, serum cortisol (evaluated through circadian rhythm and the dexamethasone suppression test for feedback inhibition function), adrenocorticotropic hormone, corticotropin-releasing hormone, and neuroimaging, is crucial for identifying OA patients who exhibit features of central sensitization and neuroendocrine dysregulation.

Management strategies are categorized into two primary types: pharmacological and non-pharmacological interventions, with the main approaches detailed in **Table 2**. Although evidence is limited, indications suggest that antidepressants and anticonvulsants are increasingly prescribed to OA patients (95). Given the scarcity of supporting evidence for OA prescriptions, caution must be exercised when prescribing these medications (96). Medications that demonstrate therapeutic effects on central sensitization include anticonvulsants, such as gabapentin and pregabalin, which act by targeting calcium channels to mitigate neuronal hyperexcitability (97); Antidepressants, specifically tricyclic antidepressants (TCAs) such as amitriptyline, inhibit the reuptake of norepinephrine and serotonin, thereby enhancing the descending inhibitory effects (97); Serotonin-norepinephrine reuptake inhibitors, like duloxetine and venlafaxine, have been shown to relieve pain associated with central sensitization. Their mechanism of action is similar to that of TCAs, but they have fewer side effects (98, 99); NMDA receptor antagonists, such as ketamine, demonstrate significant efficacy at low doses. However, caution is warranted in their use, as effectively managing side effects is crucial (100). Cannabinoid drugs possess the ability to influence the endogenous cannabinoid system, ease pain-related emotions, and are significant in the management of OA. This positions them as an interesting pharmacological target and a valuable biomarker (101).

In the context of neuroendocrine disorders, cortisol replacement and optimization are essential therapeutic strategies, particularly for patients with adrenal insufficiency who also have OA. Careful administration of these treatments is crucial to prevent the potential acceleration of joint destruction (102, 103); Alpha-2 adrenergic receptor agonists (clonidine) can reduce sympathetic nervous system activity and have analgesic and anxiolytic effects (104); Non-pharmacological interventions include transcranial magnetic stimulation (TMS), particularly repetitive TMS (rTMS), which focuses on either the motor cortex or the prefrontal cortex. This method successfully alters cortical excitability and influences pain circuits (105, 106); Transcranial direct current stimulation (tDCS) has shown the ability to modulate cortical excitability. Notably, anodal tDCS applied to the primary motor cortex (M1) has proven particularly effective in alleviating pain in patients with knee OA.

However, the influence of tDCS on functional performance seems to be limited (107). Cognitive Behavioral Therapy (CBT) empowers patients to modify specific cognitions, coping strategies, and behaviors related to pain. For instance, it aids in reducing activities such as stair climbing and prolonged sitting, effectively alleviating pain and improving functionality. This approach is especially advantageous for individuals experiencing significant central sensitization and psychosocial factors (108); Exercise therapy, defined by regular physical activity, is the cornerstone of OA treatment. Engaging in regular, appropriate, and moderate exercise can relieve joint pain and improve functional capacity. This therapeutic approach may offer benefits through multiple mechanisms, including elevating endorphin levels, regulating the HPA axis, and reducing body weight. It is crucial to develop a personalized exercise regimen tailored to the patient’s specific needs to prevent exacerbating pain due to excessive physical activity (109).

Evidence level: Pharmacological – moderate; non-pharmacological – strong. Pharmacological (antidepressants, anticonvulsants): RCTs show pain reduction in OA, particularly in centrally sensitized patients, but evidence is mainly for symptom management and quality is moderate. Caution is advised. Evidence level: moderate. Non-pharmacological (TMS, tDCS, CBT, exercise therapy): Multiple RCTs support pain relief and functional improvement; exercise therapy is a first-line core treatment. Evidence level: strong (for symptom management).

## 5 The liver-joint axis

### 5.1 NAFLD and dysregulated IGF-1 signaling contribute to systemic metabolic stress and joint pathology

The liver is an essential digestive organ in the human body, responsible for producing a variety of digestive enzymes and functioning as a central hub for numerous physiological processes (110). Research findings indicate that the Logit model regression demonstrates a significant correlation between Non-Alcoholic Fatty Liver Disease (NAFLD) and OA, with NAFLD showing a positive association with OA. Given the rising prevalence of both NAFLD and OA, it is crucial for clinicians to screen for NAFLD in patients diagnosed with arthritis and to initiate early intervention strategies (8). In addition, non-alcoholic liver disease is positively correlated with OA (8). Nevertheless, further investigation into the potential mechanisms that link liver disease and cartilage is essential.

**Figure 3:**
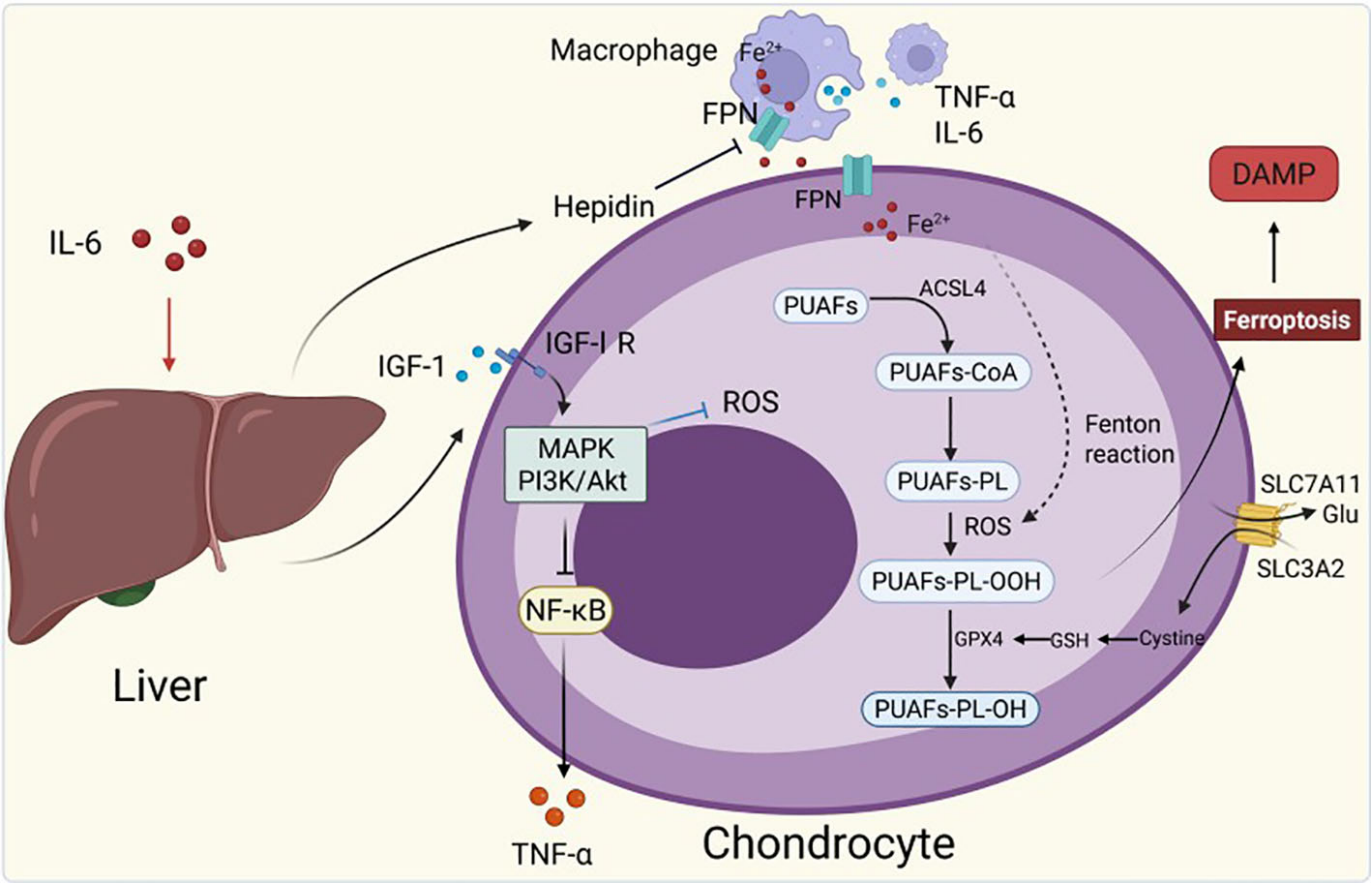
Schematic diagram of the mechanism by which the liver regulates ferroptosis in joints through iron metabolism and lipid metabolism. IGF-1 secreted by the liver acts on chondrocytes via the PI3K/AKT and MAPK signaling pathways, reducing the production of ROS and TNF-α. IL-6 induces the liver to upregulate hepcidin expression, and hepcidin inhibits FPN, suppressing iron release from macrophages and intestinal cells. This subsequently leads to iron retention within macrophages across various tissues, thereby forming an “iron-rich” microenvironment that promotes ferroptosis in joint tissues. The specific mechanism involves the following processes: ACSL4 attaches CoA moieties to long-chain PUFAs. When PUFAs are excessively attacked by ROS, LOOH are generated. During the Fenton reaction, Fe²^+^ reacts with peroxides to produce ·OH, which further amplifies the lipid peroxidation chain reaction. GPX4 can catalyze the reduction of membrane-bound PL-OOH to their corresponding non-toxic alcohols. This detoxification process relies on reduced GSH as an electron donor. When GPX4 activity decreases, or the function of System Xc^-^ (composed of SLC7A11 and SLC3A2) is impaired, GSH synthesis is reduced, leading to the failure of lipid peroxide clearance. Consequently, lipid peroxides accumulate, ultimately resulting in ferroptosis. IGF-1, Insulin-like Growth Factor-1; IL-6, Interleukin-6; FPN, ferroportin; ACSL4, Acyl-CoA synthetase long-chain family member 4; CoA, coenzyme A; LOOH, lipid hydroperoxides; Fe²^+^, ferrous ions; ·OH, hydroxyl radicals; LPCAT3, lysophosphatidylcholine acyltransferase 3; GPX4, Glutathione peroxidase 4; PL-OOH, phospholipid hydroperoxides; GSH, glutathione; SLC7A11, subunitssolute carrier family 7 member 11 Gene; SLC3A2, solute carrier family 3 member 2 Gene; PUFAs, polyunsaturated fatty acids; PL, phospholipids; ROS, Reactive Oxygen Species; DAMP, damage associated molecular patterns; TNF-α, Tumor Necrosis Factor α. **Figure 3** was created using BioRender. **Figure 3** was created using BioRender. Created in BioRender. tao, S. (2026) https://BioRender.com/61b72mb.

Insulin-like growth factor-1 (IGF-1), produced by hepatocytes, plays a crucial role in regulating cellular metabolism, proliferation, and growth across different tissues. Additionally, it affects inflammatory processes by modulating the expression of associated genes (111), These key actions of IGF-1 are detailed in **Table 1**. Research has shown that elevated serum IGF-1 concentrations are causally linked to an increased risk of OA in the hip and knee joints. Furthermore, IGF-1 may influence the progression of OA by regulating its own levels (112). Research findings indicate that IGF-1 can inhibit NF-κB signaling by modulating the MAPK and PI3K/Akt signaling pathways. **Figure 3** depicts the underlying mechanism of action. Additionally, it prevents apoptosis and suppresses OA activity by inhibiting ROS production, thus playing a protective role in the pathogenesis of OA (113).

Evidence level: Moderate (human genetic evidence plus preclinical mechanisms). The association between NAFLD and OA is based on human cross-sectional and cohort studies. The chondroprotective effects of IGF-1 via PI3K/Akt, MAPK, and NF-κB signaling have been demonstrated primarily in rabbit/rat OA models and chondrocyte cultures. Mendelian randomization studies provide human genetic evidence for a causal relationship between serum IGF-1 levels and hip/knee OA risk. Overall, the evidence is graded as moderate.

### 5.2 Iron overload and lipid peroxidation induce chondrocyte ferroptosis via the hepcidin-ferroportin axis

Systemic regulation of distant organs has gained attention in the progression of OA. The liver, a central organ for systemic iron and lipid metabolism, may significantly influence the pathological processes associated with OA by regulating ferroptosis, a novel form of cell death (114, 115). As the primary site for iron storage, the liver serves as the command center for systemic iron homeostasis. In cases of iron overload, the liver becomes a major target for damage. Hepcidin, synthesized and secreted by the liver, is the most critical hormone regulating iron metabolism. It binds to the iron export protein ferroportin, inducing its internalization and degradation, which reduces intestinal iron absorption and the release of iron from macrophages, thereby maintaining the body’s iron balance. The liver modulates the expression of hepcidin in systemic iron metabolism in response to circulating iron levels, inflammatory signals (such as IL-6), and hypoxia (116), The schematic diagram is shown in **Figure 3**. Clinical evidence supports a link between iron dysregulation and OA severity. Elevated levels of ferritin and synovial fluid iron have been reported in patients with knee OA, correlating positively with radiographic Kellgren-Lawrence grades and Western Ontario and McMaster Universities Osteoarthritis Index (WOMAC) pain scores (13, 117). Moreover, expression of the ferroptosis hallmark protein GPX4 is significantly reduced in OA cartilage compared with healthy controls, while the pro-ferroptotic enzyme ACSL4 is upregulated (13, 115). These observations suggest that ferroptosis may be actively occurring in human OA joints and contributing to disease progression (118, 119).

In systemic inflammatory conditions, OA is frequently associated with mild systemic inflammation. The liver responds to inflammatory signals, such as IL-6, by increasing the expression of Hepcidin. Elevated levels of Hepcidin inhibit the release of iron from macrophages and intestinal cells, leading to a reduction in serum iron levels. However, this mechanism also results in the retention of iron within macrophages across various tissues. Consequently, iron-overloaded macrophages may migrate to the synovial membrane of the joints or affect chondrocytes through the secretion of various factors. This interaction creates an ‘iron-rich’ microenvironment that fosters ferroptosis in the joints (117). Preclinical studies have provided direct causal evidence for ferroptosis in OA pathogenesis. In the destabilization of the medial meniscus (DMM) mouse model of OA, treatment with the specific ferroptosis inhibitor ferrostatin-1 (Fer-1) significantly attenuated cartilage erosion, reduced osteophyte formation, and preserved chondrocyte viability (13, 120). Similarly, liproxstatin-1 administration suppressed lipid peroxidation and decreased the expression of matrix-degrading enzymes MMP-13 and ADAMTS-5 in murine OA models (115). These findings demonstrate that pharmacological inhibition of ferroptosis can slow OA progression *in vivo*. Disruption of iron metabolism, particularly the accumulation of iron in joint tissues, can lead to oxidative damage and inflammation. This suggests a potential link between disorders of iron metabolism and disease progression. A key pathological mechanism involved is iron-related hypersensitivity, a form of cell death regulation driven by iron-dependent lipid peroxidation (13).

**Figure 4:**
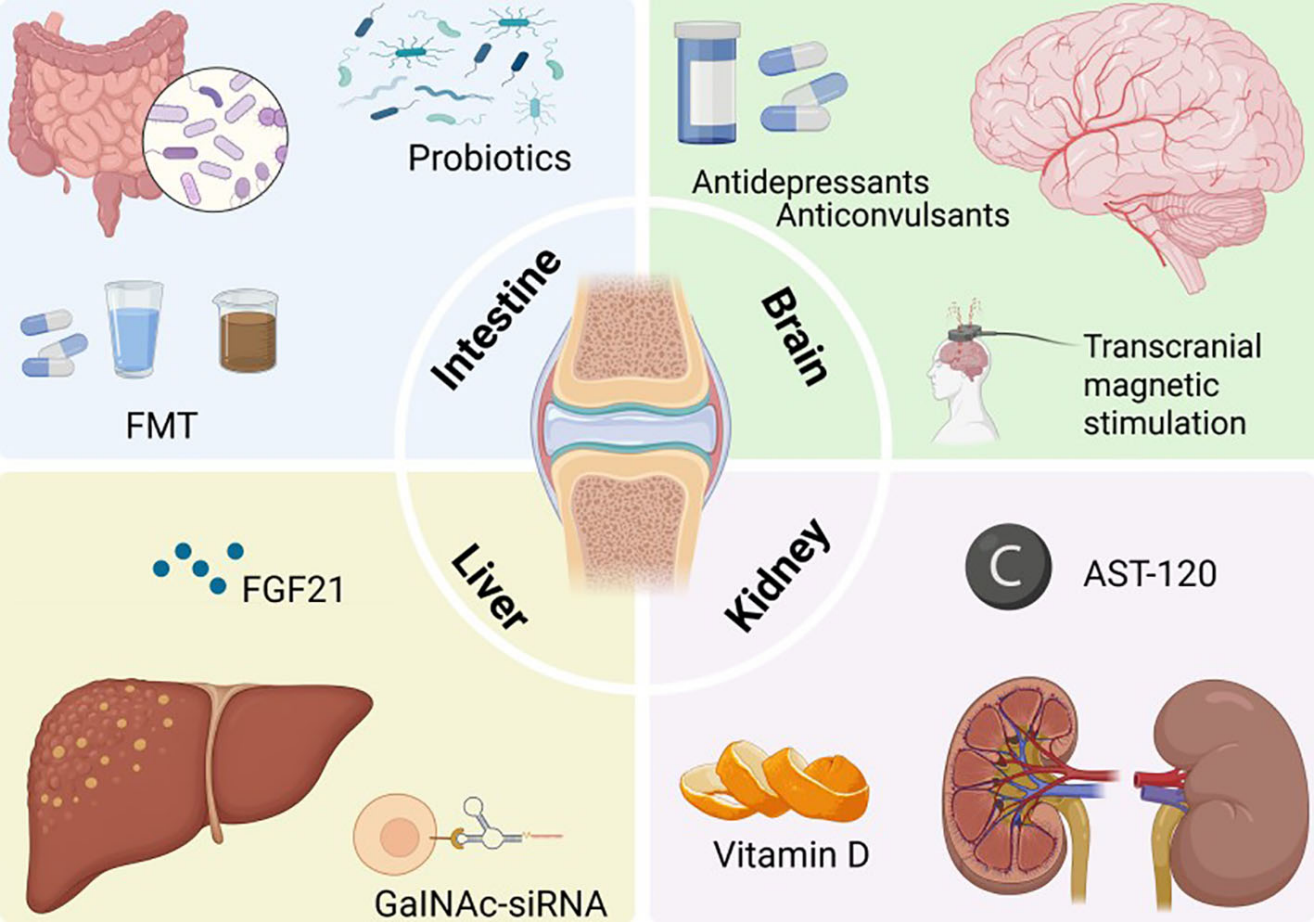
Overview of potential multi-organ targeted therapeutic strategies for OA. Treatments targeting the gut-joint axis include probiotics and FMT. Brain-joint intervention strategies consist of pharmacological therapies (antidepressants and anticonvulsants) and non-pharmacological TMS. For the liver-joint axis, therapeutic approaches involve FGF21 and GalNAc-siRNA. Kidney-joint interventions primarily rely on vitamin D supplementation and AST-120 therapy. FMT, Fecal microbiota transplantation; TMS, transcranial magnetic stimulation; FGF21, Fibroblast growth factor 21; GalNAc-siRNA, siRNA GalNAc hepatocyte delivery; AST-120, spherical carbon. **Figure 4** was created using BioRender. Created in BioRender. tao, S. (2026) https://BioRender.com/ry96h2f.

The fundamental mechanism underlying this process is the accumulation of iron, which initiates the Fenton reaction and subsequently generates ROS. This cascade of events results in lipid peroxidation, compromises cell membrane integrity, and ultimately leads to cell death. Lipid peroxidation is a critical molecular event in ferroptosis, with phospholipids (PL) abundant in polyunsaturated fatty acids (PUFAs) serving as essential components of the cell membrane. When PUFAs undergo excessive oxidative damage from ROS, they generate lipid hydroperoxides (LOOH) (121). In the Fenton reaction process, ferrous ions (Fe²^+^) react with hydrogen peroxide (H_2_O_2_) and other peroxides to generate hydroxyl radicals (·OH), which further amplify the chain reaction of lipid peroxidation (122). Glutathione peroxidase 4 (GPX4) is a selenoprotein that catalyzes the reduction of membrane-bound phospholipid hydroperoxides (PL-OOH) to non-toxic alcohols (PL-OH). This process protects the lipid bilayer and is crucial for inhibiting ferroptosis (123). This detoxification process utilizes reduced glutathione (GSH) as an electron donor, linking the function of GPX4 to the intracellular availability of cysteine. Cysteine is provided by the System Xc- transporter, which is composed of the solute carrier family 7 member 11 Gene (SLC7A11) and the solute carrier family 3 member 2 Gene (SLC3A2). This transporter enables the exchange of extracellular cysteine for intracellular glutamate (124). When GPX4 activity declines and System Xc- (SLC7A11/SLC3A2) function is impaired, the synthesis of GSH decreases, resulting in an inability to clear lipid peroxides. This leads to the accumulation of lipid peroxides and subsequent ferroptotic cell death. Acyl-CoA synthetase long-chain family member 4 (ACSL4) and lysophosphatidylcholine acyltransferase 3 (LPCAT3) are two key enzymes involved in lipid peroxidation, facilitating the esterification of PUFAs into membranes and thereby increasing sensitivity to ferroptosis. The role of ACSL4 in ferroptosis depends on its ability to attach CoA moieties to long-chain PUFAs, which are then degraded into lysophospholipids by LPCAT3 or related enzymes. Once phospholipids with PUFAs are integrated into the cell membrane, they become susceptible to peroxidation. If not cleared by GPX4, lipid peroxidation products will lead to ferroptosis (120). Genetic evidence further reinforces the role of ferroptosis in OA. Chondrocyte-specific GPX4 knockout mice spontaneously develop severe cartilage degeneration, synovitis, and osteophyte formation, phenocopying human OA (123). Conversely, overexpression of GPX4 in chondrocytes protects against DMM-induced cartilage damage (13). Additionally, systemic deletion of ACSL4 renders mice resistant to ferroptosis and ameliorates post-traumatic OA changes (120). These genetic models establish that intact ferroptosis defense mechanisms are essential for joint homeostasis. Ultimately, the accumulation of lipid peroxidation byproducts severely compromises membrane integrity, leading to irreversible cell death (125). A brief summary is presented in **Table 1**. Disruption of iron homeostasis and increased lipid peroxidation make chondrocytes and other joint cells more susceptible to oxidative damage from iron. Furthermore, the interplay between inflammation and metabolic signaling exacerbates iron hypersensitivity, creating a self-reinforcing cycle of tissue damage (13). The liver’s role as a regulator of systemic iron and lipid metabolism provides a plausible link between hepatic disorders and OA via ferroptosis. Patients with NAFLD exhibit elevated serum ferritin and hepcidin levels, as well as increased systemic oxidative stress (8). Epidemiological studies have shown a positive association between NAFLD and the prevalence of knee OA, independent of body mass index (8). Furthermore, dyslipidemia associated with hepatic steatosis may increase the delivery of polyunsaturated fatty acids to chondrocytes, enhancing their susceptibility to lipid peroxidation and ferroptosis (115, 120). Targeting the hepatic hepcidin-ferroportin axis or improving liver lipid metabolism (e.g., with FGF21 or statins) therefore represents a potential indirect strategy to suppress chondrocyte ferroptosis, as summarized in **Table 2** and **Figure 4**.

Evidence level: Preliminary to moderate (human cartilage markers exist, but causal and therapeutic evidence derives from preclinical models). Extensive preclinical evidence (*in vitro*, animal models, genetic studies) supports a pathogenic role of ferroptosis in OA. Expression changes of ferroptosis markers in human OA cartilage have been documented, but causality and therapeutic efficacy in patients remain to be established in future clinical trials. Thus, the evidence is graded as preliminary (human correlative) to moderate (preclinical causal).

### 5.3 Targeting hepatic metabolism: FGF21 and GalNAc-siRNA as emerging therapeutic strategies

Fibroblast growth factor 21 (FGF21) is primarily synthesized in the liver, brown adipose tissue, white adipose tissue, and pancreas. It is acknowledged as a vital regulator of glucose and energy metabolism, contributing to disease protection through the mediation of autophagy (126). FGF21 mitigates aging, apoptosis, and extracellular matrix degradation in OA via the SIRT1-mTOR signaling pathway, promoting autophagic flux to decrease chondrocyte apoptosis, aging, and degradation of the extracellular matrix (ECM) induced by tert-butyl hydroperoxide. Furthermore, FGF21 protects chondrocytes from apoptosis, senescence, and ECM degradation by enhancing autophagic flux, while also attenuating the progression of OA *in vivo*. These findings indicate its potential as a therapeutic agent for OA treatment (127, 128), a summary of FGF21-mediated joint regulation is provided in **Table 2**.

The asialoglycoprotein receptor (ASGPR) is expressed on hepatocyte membranes. Small nucleic acid drugs, like siRNA, require delivery systems for improved efficiency and targeting. The GalNAc (N-acetylgalactosamine) conjugation is the most common siRNA delivery system. GalNAc binds to ASGPR, allowing the GaINAc-siRNA (siRNA GalNAc hepatocyte delivery) conjugate to specifically target the receptor. This interaction facilitates siRNA transport into the cell via endocytosis, enabling targeted delivery to hepatocytes (129). The application of siRNA has emerged as a promising strategy for the treatment of OA due to its capacity to precisely target specific genes. siRNA molecules can regulate post-transcriptional gene expression by modulating key pathways associated with cell proliferation, apoptosis, senescence, autophagy, biomacromolecule secretion, inflammation, and bone remodeling (130, 131).

Evidence level: Preliminary (both strategies are at preclinical stage with no human OA data). FGF21 therapy: In animal models, it promotes chondrocyte autophagy and reduces senescence/ECM degradation via SIRT1-mTOR signaling. No human OA data. GalNAc-siRNA liver-targeted delivery: Platform established, but in OA it remains a conceptual proposal with no preclinical or clinical OA data. Both strategies are graded as preliminary/emerging and require translational validation.

## 6 The kidney-joint axis

### 6.1 Chronic kidney disease drives joint degeneration through indoxyl sulfate and FGF23-Wnt signaling

A survey study revealed that 53.9% of patients with chronic kidney disease undergoing long-term hemodialysis were diagnosed with OA. This finding highlights the potential link between chronic kidney disease and OA (9). Uremic toxins, including indoxyl sulfate (IS) and p-cresyl sulfate (PCS), are recognized risk factors for disorders associated with chronic kidney disease (CKD), particularly affecting bone and joint metabolism. Both IS and PCS are classified as protein-bound uremic toxins (132). The removal of these substances through dialysis is difficult. Furthermore, patients undergoing dialysis often develop conditions such as arthritis, osteoporosis, and fractures. These complications may be related to abnormalities in bone metabolism, inflammation, and age-related changes induced by IS and PCS (133).

The connection between chronic kidney disease and OA is complex, primarily due to the accumulation of IS and PCS, which promote bone resorption and inhibit parathyroid hormone receptor expression in osteoblasts. These compounds also increase blood calcium levels and reduce bone density (133, 134).

**Figure 5:**
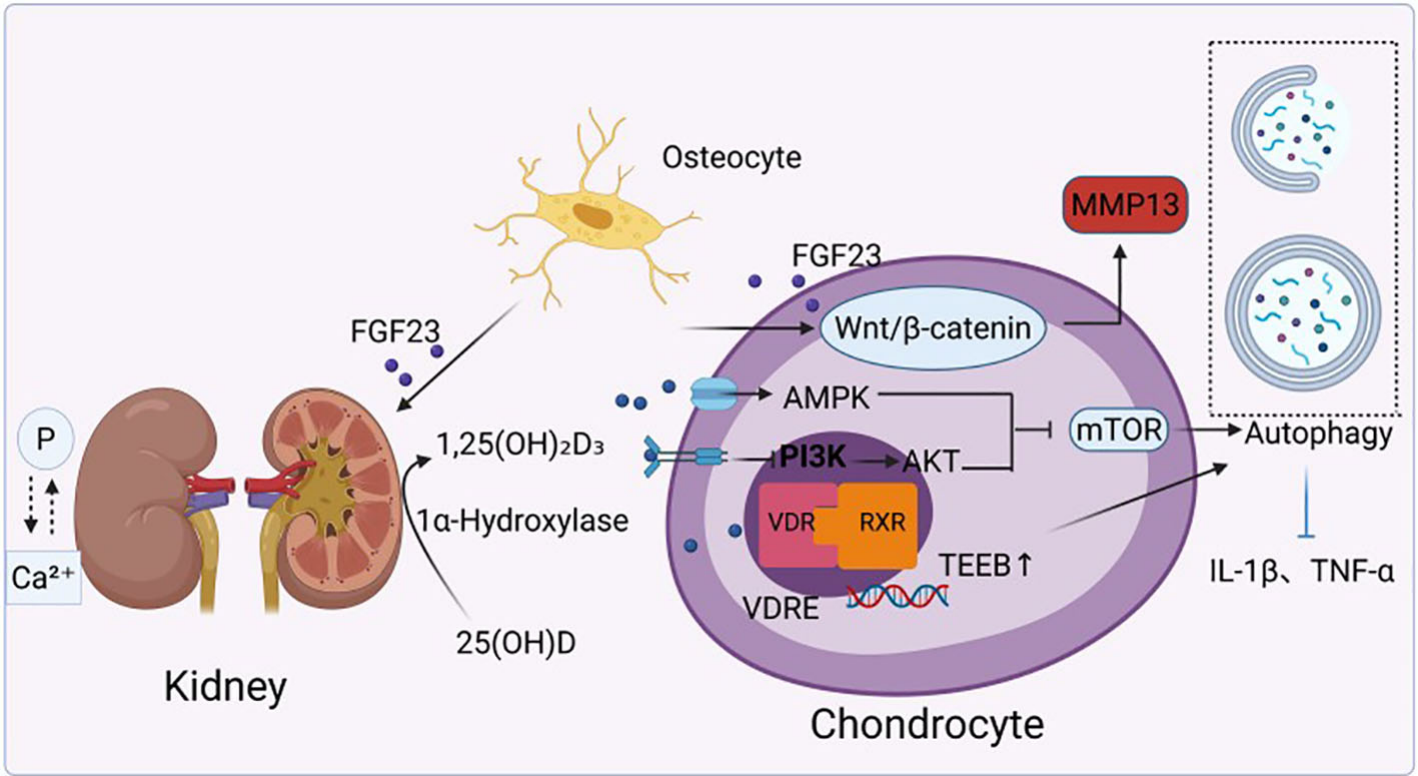
Schematic diagram illustrating the mechanisms by which Vitamin D and FGF23 regulate joint health. FGF23 is a key factor linking bone metabolism to renal calcium-phosphate metabolism by regulating serum phosphate levels. It modulates the Wnt/β-catenin signaling pathway in chondrocytes, upregulates the expression of ColX, MMP13, and MMP9, and ultimately promotes OA progression. Vitamin D, activated by 1α-hydroxylase, enhances the fusion of autophagosomes and lysosomes through VDR/RXR-mediated upregulation of TFEB expression, thereby alleviating inflammatory cell death. Additionally, vitamin D activates the AMPK/mTOR pathway and inhibits the PI3K/AKT/mTOR pathway, promoting autophagy and reducing the production of pro-inflammatory cytokines. FGF23, fibroblast growth factor 23; MMP13, Matrix Metallopeptidase 13; VDR, vitamin D receptor; RXR, retinoid X receptor; TEEB, transcription factor EB; IL-1β, Interleukin—1β; TNF-α, Tumor Necrosis Factor α. **Figure 5** was created using BioRender. Created in BioRender. tao, S. (2026) https://BioRender.com/tnbi7bc.

In patients with CKD across all stages, elevated levels of fibroblast growth factor 23 (FGF23) are significantly associated with adverse outcomes (135). Although research on the effects of kidneys and CKD on cartilage is limited, FGF23 has emerged as a vital factor connecting bone metabolism with renal calcium and phosphate regulation. FGF23 is a hormone that is primarily secreted by osteocytes (135). It regulates serum phosphate levels by suppressing the expression of renal phosphate transport proteins in the proximal renal tubule, thereby maintaining the equilibrium between bone synthesis and metabolism. Recent studies have increasingly clarified the relationship between fibroblast growth factor (FGF) and arthritis, providing evidence that FGF23 enhances the expression of collagen type X, MMP13, and MMP9. This modulation ultimately contributes to the progression of OA through its influence on the Wnt/β-catenin signaling pathway in chondrocytes (136, 137), **Figure 5** illustrates the aforementioned content. Furthermore, research has demonstrated that FGF23 plays a role in maintaining the chondrocyte phenotype in OA (137). The kidneys play a vital role in the regulation of FGF23 by managing calcium and phosphorus metabolism. Through various protein signaling pathways, they enhance the expression of MMPs, which ultimately influences OA.

Evidence level: Moderate (human observational data plus preclinical mechanisms). Cross-sectional surveys document a higher prevalence of OA in CKD patients. The effects of uremic toxins (IS, PCS) on bone metabolism have been investigated in cellular and animal experiments. Evidence that FGF23 regulates Wnt/β-catenin signaling and induces MMP13/9 expression comes primarily from *in vitro* human OA chondrocyte cultures and mouse models. Large-scale prospective human studies supporting a causal relationship between FGF23 and OA are lacking. Thus, the evidence is graded as moderate (human observational plus preclinical mechanisms).

### 6.2 Vitamin D deficiency impairs chondrocyte autophagy and promotes inflammatory cell death

The kidneys serve as the primary site for the activation of Vitamin D, converting it into the biologically active steroid hormone 1,25-(OH)_2_D_3_. This hormone is essential for maintaining bone health, as it boosts intestinal calcium absorption, minimizes renal calcium excretion, promotes bone mineralization, and aids in bone remodeling. Furthermore, vitamin D is involved in regulating key cellular processes such as differentiation, proliferation, apoptosis, and angiogenesis. The vitamin D receptor (VDR), which is abundantly present in the parathyroid glands, intestines, kidneys, and bones, plays a crucial role in maintaining the balance of calcium and phosphorus. Beyond its traditional metabolic functions, VDR signaling within the lymphatic system also plays a significant role in modulating systemic immunity.

Studies have demonstrated that plasma levels of 25(OH)D are significantly lower in OA patients (14). Furthermore, vitamin D deficiency in plasma prior to the age of 60 is significantly associated with a higher prevalence of OA. A positive correlation has been established between plasma vitamin D deficiency and knee OA, indicating that low levels of vitamin D may increase the risk of developing knee OA. This suggests that a deficiency of 25(OH)D is a critical risk factor for this condition (138). Vitamin D is strongly associated with OA. Appropriate supplementation of vitamin D can alleviate pain and inflammation related to OA, improve bone quality in patients, and relieve joint discomfort. Furthermore, studies have demonstrated that vitamin D (the active form 1,25(OH)_2_D_3_) can activate the AMPK signaling pathway, inhibiting the mTOR pathway, thereby alleviating the mTOR-mediated suppression of autophagy and initiating the autophagy process (139). Autophagy is a cellular self-degradation process that removes damaged organelles, proteins, and other components, maintaining cellular homeostasis. Enhanced autophagy promotes the removal of damaged organelles, reduces apoptosis, and inhibits the release of inflammatory factors like IL-1β and TNF-α. In knee OA (KOA), decreased autophagy levels are closely linked to increased chondrocyte apoptosis and worsened inflammation (139).

Vitamin D can also counteract its inhibitory effect on autophagy by suppressing the PI3K/AKT/mTOR pathway, promoting autophagosome formation, and reducing arthritis inflammation and cartilage destruction (140). The vitamin D action pathway is illustrated in **Table 1**. Vitamin D (1,25-(OH)_2_D_3_) crosses the nuclear membrane and binds to the VDR within the nucleus. The VDR forms a heterodimer with the retinoid X receptor (RXR), leading to the formation of the VDR/RXR complex. This complex specifically binds to DNA sequences in the promoter region of the transcription factor EB (TFEB) gene, known as the vitamin D response element. Consequently, this binding directly upregulates TFEB gene transcription and translation, leading to an increased total amount of nuclear TFEB protein within the cell. This process promotes lysosome biogenesis by enhancing TFEB and boosts autophagic degradation. In KOA chondrocytes, vitamin D increases TFEB expression, facilitates autophagosome-lysosome fusion, and reduces inflammatory cell death (140, 141), the autophagy regulation of OA by vitamin D is shown in **Figure 5**. Vitamin D promotes autophagy through the AMPK/mTOR pathway and works in concert to protect joints by decreasing oxidative stress and enhancing TFEB-mediated lysosomal function. This dual action helps to preserve chondrocyte health and slows the advancement of KOA. The fundamental mechanism at play is the restoration of autophagy homeostasis, which disrupts the destructive cycle of “apoptosis-inflammation-oxidative damage”. Nevertheless, the clinical evidence for vitamin D supplementation in OA remains controversial and requires critical appraisal. The most definitive evidence to date comes from a large randomized controlled trial by McAlindon et al. (2013), which found that vitamin D supplementation over two years did not significantly reduce knee pain or cartilage volume loss compared with placebo in patients with symptomatic knee OA (142). This null finding stands in apparent contrast to the mechanistic evidence described above, which suggests plausible biological pathways through which vitamin D could protect chondrocytes.

Several explanations may account for this discrepancy. First, the beneficial effects of vitamin D may be conditional on baseline vitamin D status; supplementation in already-replete individuals may not yield additional benefits. Second, the two-year follow-up period may be insufficient to detect structural changes in cartilage, given the slow progression of OA. Third, patient stratification by vitamin D receptor polymorphisms or other genetic factors might identify subgroups that respond differently. Fourth, the dose and formulation of vitamin D (daily versus intermittent, D_2_ versus D_3_) may influence outcomes. Thus, while vitamin D remains a biologically plausible target for OA intervention based on its role in autophagy regulation and anti-inflammatory effects (139), current high-quality RCT evidence does not support its routine use as a disease-modifying agent for OA. Future trials should focus on vitamin D-deficient populations and incorporate longer follow-up periods and advanced imaging endpoints to clarify whether supplementation provides benefit in specific patient subsets.

Evidence level: Conflicting (strong preclinical mechanisms, but high-quality RCTs show no structural benefit). Multiple cross-sectional and cohort studies support an association between vitamin D deficiency and OA prevalence/severity. Evidence that vitamin D promotes autophagy and suppresses inflammation via VDR/RXR-TFEB, AMPK/mTOR, and PI3K/Akt/mTOR pathways derives primarily from *in vitro* OA chondrocytes and animal models (preclinical). However, human RCTs (e.g., McAlindon et al.) have yielded inconsistent effects on structural improvement, with the largest trial showing null results. Thus, the evidence is graded as conflicting (strong preclinical rationale but conflicting clinical evidence), and further stratified studies are needed.

### 6.3 Vitamin D supplementation and uremic toxin clearance as therapeutic interventions

The traditional treatment methods for OA include exercise, physical therapy, analgesics, and surgical interventions, such as joint replacement. However, innovative treatment strategies for CKD and OA continue to emerge, some of which may also be applicable to CKD-related OA. It is important to note that there is currently no definitive treatment for OA. Certain pharmacological agents that alleviate CKD may also mitigate the effects of toxin production.

For the management of CKD-OA, AST-120 is primarily indicated for patients with CKD stages 3–5 (non-dialysis dependent) and elevated serum levels of protein-bound uremic toxins, with no specific restriction on OA grade (134, 143). In terms of outcomes, AST-120 reduces uremic toxin levels, slows CKD progression, and improves CKD-related bone metabolism abnormalities, but no direct evidence supports its ability to alleviate OA structural damage or symptoms. The administration route is systemic (oral), taken three times daily after meals (144, 145).

NSAIDs are mainly indicated for moderate-to-severe OA pain. However, their use in CKD patients must be strictly restricted: only for CKD stages 1–2 (eGFR ≥60 mL/min/1.73m²) with short-term oral use; for CKD stages 3–5, oral NSAIDs are generally contraindicated, while topical formulations may be considered with caution. In terms of efficacy, NSAIDs effectively relieve OA pain and improve joint function, but long-term or high-dose use can lead to reduced eGFR, acute kidney injury, CKD progression, interstitial nephritis, and other nephrotoxic events. Administration routes include systemic (oral) and topical (gels/patches). Topical NSAIDs (e.g., diclofenac gel) have lower systemic absorption and relatively lower renal risk, making them a potential alternative for patients with CKD stage 3, though renal function monitoring remains necessary (146).

Vitamin D supplementation improves serum vitamin D levels, enhancing pain relief, joint function, and inflammatory markers in patients with KOA. The summary of CKD-OA is presented in **Table 2**. Furthermore, it is significantly associated with the progression of OA, particularly in patients with vitamin D deficiency, as indicated by a reduction in cartilage volume (147).

Some studies suggest that vitamin D supplementation does not significantly improve the structure of KOA. This lack of effect may relate to factors such as dosage, duration of supplementation, and the individual patient’s level of OA (142). Further research is necessary to establish the optimal therapeutic dosage. While the mechanism is well understood, clinical application still requires an assessment of the patient’s vitamin D status. Furthermore, investigations into this axis are still in their early stages, and the available evidence is not as robust as that for other axes. Consequently, additional basic and clinical studies are needed in the future.

Evidence level: **AST-120:** This agent adsorbs uremic toxin precursors in CKD and may alleviate CKD-related bone disorders. However, no study has investigated its use in OA. Therefore, the evidence level for OA is **preliminary**, based solely on the CKD context. **Vitamin D supplementation:** Multiple RCTs have demonstrated symptomatic benefits, including pain relief and functional improvement. However, the largest RCT (McAlindon et al.) found no structural cartilage benefit. The efficacy may depend on baseline vitamin D status. Consequently, the evidence level for vitamin D is **conflicting**: moderate evidence supports symptomatic improvement, but no evidence supports structural modification.

## 7 Coordinated crosstalk among gut, brain, liver, and kidney forms a multi-organ regulatory network in OA

### 7.1 The gut-brain-joint axis integrates neural, immune, and metabolic signals to modulate pain and inflammation

The brain–gut axis is a bidirectional communication network linking the brain and the gut that integrates information through neural, immune, metabolic, and endocrine pathways, thereby indirectly shaping the initiation and progression of arthritis. In particular, the vagus nerve serves as a core conduit for brain–gut signaling: gut microbiota and their metabolites can activate vagal afferent fibers, relaying signals to the nucleus tractus solitarius and higher brain regions, which in turn modulate systemic inflammation, stress responses, and pain perception (148, 149); Conversely, under stress, the brain can act through the autonomic nervous system to alter gut motility, mucus secretion, and local immune status, thereby reshaping gut microbiota composition and intestinal barrier function (150).

Furthermore, metabolites produced by the gut microbiota, such as short-chain fatty acids, serotonin, and GABA, can influence central nervous system function via the bloodstream or the vagus nerve, and can also modulate arthritis-related behaviors and pain perception (151). Notably, the host circadian system and the gut microbiota are reciprocally regulated: clock genes drive rhythmic oscillations in the microbiota, whereas alterations in the microbiota can, in turn, feedback to modulate central circadian gene expression. This bidirectional disruption is closely tied to metabolic dysregulation and heightened inflammation in arthritis, particularly in osteoarthritis and rheumatoid arthritis (152). Taken together, by integrating immune, metabolic, and neural signals, the brain–gut axis serves as a critical link in the brain–gut–joint axis, bridging central and peripheral inflammation.

### 7.2 The gut-liver-joint axis orchestrates bile acid metabolism and systemic inflammation

The gut–liver axis facilitates bidirectional metabolic crosstalk between the gastrointestinal tract and the liver in both health and disease. The hepatic portal vein collects blood from the small intestine, colon, spleen, and pancreas, serving as a hallmark anatomical conduit for gut–liver communication. Nutrients, microbial antigens, metabolites, and bile acids modulate metabolic and immune responses in the gut and liver, and these interactions, in turn, reciprocally shape the structure and function of the gut microbiota (153). By metabolizing bile acids, the gut microbiota can engage in a range of host regulatory processes and activate innate immune genes in the small intestine, thereby directly or indirectly shaping the gut microbiota itself (154). Gut microbiota dysbiosis and intestinal barrier dysfunction can lead to systemic microbial translocation into the hepatic portal circulation, resulting in reduced secretion of various bile acids. Gut microbial metabolites (e.g., LPS, SCFAs) and liver-derived products (e.g., bile acids, hepcidin) engage in reciprocal crosstalk via the portal circulation, collectively shaping systemic metabolic and inflammatory tone (155, 156).

Bile acid metabolism along the gut–liver axis is primarily governed by FXR and G protein-coupled bile acid receptors. These receptors have already been used clinically for the treatment of liver-related diseases. As research has progressed, the therapeutic effects of FXR and TGR5 in osteoarthritis have also been uncovered and exploited. Notably, the clinically approved drug UDCA has been shown to alleviate osteoarthritis and prevent cartilage degradation in mice by acting through the intestinal FXR–joint GLP-1 axis (10); Activation of the bile acid receptor TGR5 ameliorates IL-1β-induced chondrocyte senescence and further inhibits the degradation of type II collagen and aggrecan in human chondrocytes (47, 49). Although clinical evidence remains limited, therapeutic strategies targeting multiple organ systems are gradually expanding. In metabolic diseases, dysbiosis directly increases intestinal permeability to bacterial products, accompanied by an expansion of pro-inflammatory bacteria and a depletion of beneficial microbes, thereby elevating the systemic levels of these products and accelerating disease progression.

### 7.3 The gut-kidney-joint axis drives comorbid progression of chronic kidney disease and osteoarthritis

Gut dysbiosis drives the comorbid progression of CKD and OA through the gut–kidney–joint axis. In CKD, aberrant microbial metabolism leads to the accumulation of uremic toxins, which directly inhibit osteoblast differentiation and promote renal fibrosis (157, 158). In OA, the gut microbiota is characterized by an expansion of pro-inflammatory bacteria and a reduction in butyrate-producing anti-inflammatory bacteria. These shifts are driven by dysregulation of the tryptophan metabolic pathway, including activation of the indoleamine 2,3-dioxygenase (IDO) pathway (159)and endotoxin-mediated inflammatory responses in synovial macrophages (17), exacerbating articular cartilage degeneration and pain. Furthermore, both CKD and OA exhibit a shared dysbiotic module characterized by the expansion of pro-inflammatory and endotoxin-producing bacteria, depletion of SCFA-producing beneficial bacteria, and disruption of the intestinal barrier (160). Thus, targeting the gut microbiota through dietary fiber, probiotics, and prebiotics may represent a promising strategy for concurrently improving renal function and joint health (161).

No studies have examined whether kidney-derived uremic toxins, such as indoxyl sulfate, cross the blood–brain barrier and contribute to central sensitization. Likewise, it remains unknown whether liver-derived FGF21 can traverse the blood–brain barrier to modulate pain perception. These inter-organ interactions represent a critical and largely uncharted frontier. To address these gaps, we propose the following strategies for future investigation: (1) multi-organ co-culture microfluidic chips that recapitulate the gut–liver–brain–kidney axis; (2) conditional gene-knockout animals in which specific pathways are disrupted in distinct organs to assess OA phenotypic changes; and (3) longitudinal multi-omics cohort studies that integrate plasma metabolomics, fecal metagenomics, and neuroimaging to identify synergistic cross-organ biomarkers.

## 8 Discussion

This review systematically highlights the essential function of the gut-brain-liver-kidney axis in OA. Dysbiosis of the gut microbiota and its metabolic byproducts contribute to systemic inflammation. Additionally, central sensitization in the brain is a primary mechanism underlying the maldaptive pain experience in OA. Unlike the direct catabolic effects of gut dysbiosis or chondrocyte ferroptosis, CS amplifies pain signals and perpetuates a chronic pain state. This state, in turn, drives physical inactivity and altered biomechanics, which can secondarily accelerate joint degeneration, forming a destructive feedback loop. In the liver, the disruption of lipid and iron metabolism fosters chondrocyte ferroptosis. Furthermore, the kidneys play a crucial role in maintaining bone-cartilage homeostasis through vitamin D metabolism and the effects of uremic toxins. Given these interconnected mechanisms, we anticipate novel therapeutic strategies that target each component of the organ axis.

Importantly, as discussed in Section 7, these organ systems do not operate in parallel isolation; rather, they are interconnected through feedforward and feedback loops. For instance, in addition to gut-derived LPS triggering joint inflammation, gut dysbiosis elevates pro-inflammatory cytokines and, via vagal afferent signaling, promotes central sensitization. Appreciating this multi-organ crosstalk is essential for moving beyond a single-axis perspective and truly understanding OA at a systems level.

**Figure 4** graphically presents the main therapeutic approaches outlined previously, aiming to shift the research and treatment of OA from a localized symptomatic approach to a systemic etiology-oriented paradigm.

The conceptualization of OA as a systemic disorder mediated by multi-organ communication presents significant challenges in mechanistic, translational, and clinical domains. Mechanistically, understanding the complex and dynamic interactions between organ systems faces two primary obstacles: the technical difficulty of obtaining long-term, parallel physiological recordings across varying states (162) and the absence of mature analytical and computational frameworks capable of interpreting the effects of these multi-organ influences on OA pathogenesis from continuous data streams (163). Furthermore, a notable knowledge gap remains concerning the synergistic or antagonistic interactions between metabolites and inflammatory mediators originating from various organs. This uncertainty renders many of the proposed mechanistic networks speculative and emphasizes the necessity for longitudinal validation (164). In translational medicine, several significant bottlenecks impede progress. Interventions like FMT offer compelling experimental evidence supporting the gut-joint axis (165), their application in establishing human causality is constrained (67). Ethical considerations, variable efficacy in modulating established dysbiosis, and the impracticality of long-term FMT studies for chronic disease investigation pose substantial limitations (67). Although human microbiota-associated (HMA) rodent models are conceptually valuable for causal inference, their translational relevance is often limited by their inability to replicate critical human ecological factors, such as diet, lifestyle, and host genetics, that contribute to disease phenotypes in humans (166). This limitation complicates establishing definitive causal links between microbiome alterations and OA pathogenesis. The complexity of clinical trial design for systemic OA therapeutics is a significant challenge (167, 168). The conventional ‘one disease, one target’ drug development paradigm is ill-suited for a condition that involves multiple organ systems (168). Trial design is inherently complicated by the need for multidisciplinary collaboration, the complex assessment of pharmacodynamics and pharmacokinetics for organ-targeted therapies, and the potential for antagonistic effects between drugs that target different systems (169). Additional hurdles arise from the considerable challenge of monitoring the spatiotemporal distribution of crucial metabolites across various organ compartments, as traditional single-time-point assays fall short. Moreover, there is no consensus on the most effective clinical endpoints; conventional structural and pain metrics may not accurately capture systemic regulatory effects, while innovative multidimensional biomarkers necessitate further validation. Finally, the need for patient stratification based on organ-specific phenotypes adds further complexity to the design and execution of trials (167, 168).

To address the current challenges, future research should prioritize the following areas: Our primary goal is to integrate multi-omics data within the precision medicine framework. By incorporating various dimensions of data—such as genomics, transcriptomics, proteomics, metabolomics, and microbiomics—we aim to systematically unravel the heterogeneity of OA and pinpoint disease subtypes characterized by unique pathological mechanisms and clinical phenotypes. Building on this foundation, the combination of network-based drug positioning with pharmacological approaches will facilitate the systematic identification of potential therapeutic compounds or biologics that can target multiple organs and pathways. This approach aims to transition OA treatment from a “single-target” model to a “network-based regulation” model, thereby providing a robust theoretical foundation for personalized therapy (163, 170, 171). Develop targeted delivery and microenvironment modulation strategies. Utilize delivery systems like nanomaterials and engineered exosomes for organ-specific and cell-specific delivery of drugs, nucleic acids, or bioactive molecules, enhancing therapeutic efficacy and reducing systemic side effects (172, 173). Moreover, as the largest organ in the human body, the skin has been shown by recent studies to accelerate OA progression when aged (174). Transdermal drug delivery systems demonstrate efficacy comparable to intra-articular injections while avoiding the discomfort and risks associated with repeated joint injections. More importantly, since the drug primarily acts locally within the skin, systemic exposure is minimal, resulting in a favorable safety profile (175). In the future, integrating these systems with innovative materials presents exciting opportunities for creating a variety of safe and effective therapeutic strategies. Increasing evidence suggests that mitochondrial dysfunction plays a crucial role as a pathological factor in the onset and advancement of OA (176). The abnormal release of mitochondrial metabolites and components can initiate inflammatory cascades, lead to oxidative stress, and ultimately cause cell death, which in turn accelerates joint degeneration. Therefore, interventions aimed at targeting mitochondria that influence mitochondrial quality control systems—such as mitophagy and mitochondrial biogenesis—or metabolic pathways are anticipated to represent a promising therapeutic strategy for decelerating disease progression (177, 178)—finally, advanced regenerative medicine and biotherapeutic strategies. Stem cell therapies, particularly those involving mesenchymal stem cells (MSCs), have shown significant potential in promoting cartilage repair and modulating the local immune microenvironment in intra-articular injection therapies. This efficacy is attributed to their multidirectional differentiation capabilities and immunomodulatory properties (179, 180). Future research should optimize cell sources, delivery methods, and pretreatment strategies, and their interaction with the host microenvironment. Large-sample clinical studies with long-term follow-up are needed to clarify safety, efficacy, and durability. In addition, technologies like gene editing and tissue engineering are expected to offer new solutions for joint structural repair (181, 182).

However, it is important to recognize that, despite the promising potential of various research directions, many remain at the preclinical or early clinical exploration stages. Moving forward, there is an urgent need for well-designed clinical trials that include long-term follow-up to gather high-quality clinical evidence and develop a reliable biomarker system for effective patient stratification. Meanwhile, interdisciplinary collaboration—encompassing fields such as bioengineering, computational biology, and clinical medicine—will be crucial for translating these innovative strategies from theoretical frameworks into practical clinical applications. The ultimate aim is to shift the treatment paradigm for OA from merely managing symptoms to implementing active, systems biology-based disease modification and personalized health management.

A critical caveat to the framework proposed herein is the distinction between correlation and causation. While we have highlighted compelling associations between systemic dysregulation and OA, the current evidence largely supports a modulatory rather than an initiatory role for the gut-brain-liver-kidney axis. It remains plausible that primary, joint-localized pathology-–driven by mechanical factors-–is the initial event, and that systemic inflammation or metabolic disturbances subsequently act as accelerators. Future longitudinal studies and mechanistic trials are urgently needed to establish the temporal sequence and causal direction of these multi-organ interactions.

## 9 Conclusion

OA is increasingly recognized as a systemic disorder arising from complex multi-organ interactions, rather than merely a localized joint disease. Distal organs—including the gut, liver, and kidney contribute to OA pathogenesis through direct structural pathways involving systemic inflammation, metabolic disorders, and cell death. In contrast, the brain primarily regulates the pain dimension of the disease through central sensitization. Targeting these systemic axes presents promising novel therapeutic avenues that extend beyond conventional symptomatic relief. However, the field faces significant challenges, including a lack of direct mechanistic evidence in humans, the complexity of long-term clinical validation, and the necessity for innovative trial designs for multi-target therapies. Future research should focus on clarifying the causal roles of specific organ systems in OA, identifying dependable biomarkers for patient stratification, and translating these findings into effective, personalized treatment strategies.

Overcoming these obstacles will be crucial for advancing from a conceptual understanding of systemic regulation to tangible clinical breakthroughs in OA management.
